# Molecular determinants of inhibition of the human proton channel hHv1 by the designer peptide C6 and a bivalent derivative

**DOI:** 10.1073/pnas.2120750119

**Published:** 2022-06-01

**Authors:** Ruiming Zhao, Rong Shen, Hui Dai, Eduardo Perozo, Steve A. N. Goldstein

**Affiliations:** ^a^Department of Physiology and Biophysics, School of Medicine, Susan and Henry Samueli College of Health Sciences, University of California, Irvine, CA 92697;; ^b^Department of Biochemistry and Molecular Biology, Gordon Center for Integrative Science, University of Chicago, Chicago, IL 60637;; ^c^Department of Pediatrics, School of Medicine, Susan and Henry Samueli College of Health Sciences, University of California, Irvine, CA 92697;; ^d^Department of Pharmaceutical Sciences, School of Pharmacy and Pharmaceutical Sciences, Susan and Henry Samueli College of Health Sciences, University of California, Irvine, CA 92697

**Keywords:** voltage-gated proton channel, C6, tethered toxin, bivalent toxin, molecular dynamics simulations

## Abstract

The human voltage-gated proton channel (hHv1) is important for control of intracellular pH. We designed C6, a specific peptide inhibitor of hHv1, to evaluate the roles of the channel in sperm capacitation and in the inflammatory immune response of neutrophils [R. Zhao *et al.*, *Proc. Natl. Acad. Sci. U.S.A.* 115, E11847–E11856 (2018)]. One C6 binds with nanomolar affinity to each of the two S3–S4 voltage-sensor loops in hHv1 in cooperative fashion so that C6-bound channels require greater depolarization to open and do so more slowly. As depolarization drives hHv1 sensors outwardly, C6 affinity decreases, and inhibition is partial. Here, we identified residues essential to C6–hHv1 binding by scanning mutagenesis, five in the hHv1 S3–S4 loops and seven on C6. A structural model of the C6–hHv1 complex was then generated by molecular dynamics simulations and validated by mutant-cycle analysis. Guided by this model, we created a bivalent C6 peptide (C6_2_) that binds simultaneously to both hHv1 subunits and fully inhibits current with picomolar affinity. The results help delineate the structural basis for C6 state-dependent inhibition, support an anionic lipid-mediated binding mechanism, and offer molecular insight into the effectiveness of engineered C6 as a therapeutic agent or lead.

## Significance

We designed C6 peptide to address the absence of specific inhibitors of human voltage-gated proton channels (hHv1). Two C6 bind to the two hHv1 voltage sensors at the resting state, inhibiting activation on depolarization. Here, we identify the C6–hHv1 binding interface using tethered-toxin variants and channel mutants, unveil an important role for negatively charged lipids, and present a model of the C6–hHv1 complex. Inspired by nature, we create a peptide with two C6 epitopes (C6_2_) that binds to both channel subunits simultaneously, yielding picomolar affinity and significantly improved inhibition at high potentials. C6 and C6_2_ are peptides designed to regulate hHv1, a channel involved in innate immune-system inflammatory pathophysiology, sperm capacitation, cancer-cell proliferation, and tissue damage in ischemic stroke.

Human voltage-gated proton channels (hHv1s) are expressed in many human tissues, including innate and adaptive immune cells, cancer cells, and sperm (1, 2). hHv1 channels comprise two identical subunits, each with 273 residues and 4 transmembrane spans (S1–S4), that resemble the voltage-sensor domains (VSDs) in conventional voltage-gated ion channels (3, 4). In hHv1s, there are two H^+^-selective conduction pathways, one in each VSD (5). The activation of hHv1 depends on both the transmembrane potential and the pH gradient across the membrane (1). Upon membrane depolarization, hHv1 S4 segments move outward, leading to conformational changes that open the H^+^ conduction pathways (1).

hHv1 has been implicated in many aspects of health and disease (1, 2). In the absence of known high-affinity and specific inhibitors of the channel, we designed a selective inhibitor of hHv1, the C6 peptide, and used it to demonstrate that H^+^ efflux via the channel is required in human sperm to induce intracellular alkalization and Ca^2+^ influx to initiate capacitation. Furthermore, we showed that it also operates in human neutrophils to maintain cytoplasmic pH during the respiratory burst, allowing sustained reactive oxygen species (ROS) production (6, 7). We identified C6 using a high-throughput, phage-display strategy whereby ∼1 million novel peptides were fabricated on an inhibitor cysteine knot (ICK) toxin scaffold and sorted on purified hHv1 protein. Phagemids expressing C6 were selected by their capacity to bind to hHv1 protein (6). C6 has 41 residues, including 6 cysteines, that form three intramolecular disulfide bonds (*SI Appendix*, Fig. S1).

**Fig. 1.**
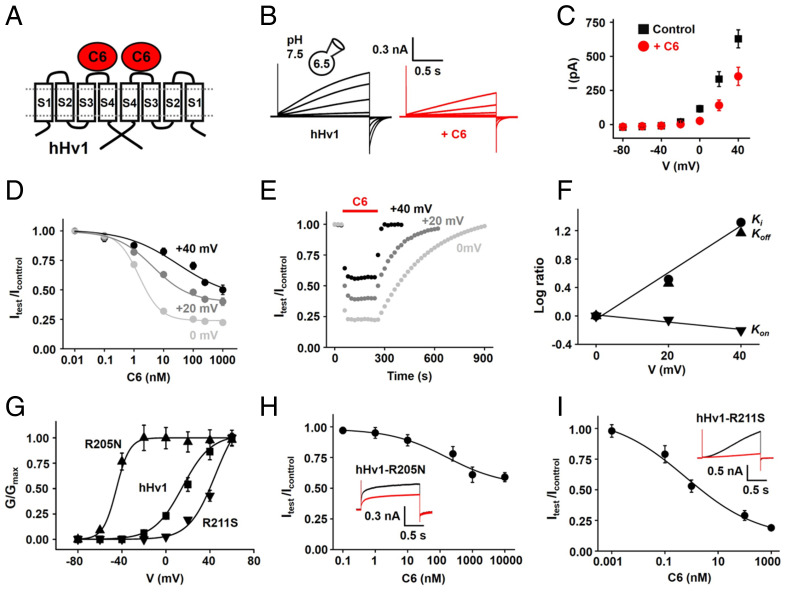
State-dependent inhibition of C6 on hHv1 channels. hHv1, hHv1-R205N, and hHv1-R211S channels were expressed in HEK293T cells and studied by whole-cell patch clamp to assess blocking parameters with a holding voltage of −60 mV, 1.5-s test pulses, and 10-s interpulse intervals, with pH_i_ = 6.5 and pH_o_ = 7.5. Values are mean ± SEM; *n* = 3 to 6 cells for each condition. (*A*) Cartoon showing two C6 peptides binding on two subunits of a hHv1 channel. (*B*) Representative H^+^ current traces for hHv1 channels before (*Left*) and in the presence of 250 nM C6 (*Right*) with steps of 20 mV from −60 to +40 mV. The peak current at the end of the step was used to determine the extent of block. (*C*) Current–voltage relationships for hHv1 in the absence (black) or presence (red) of 250 nM C6. C6 inhibition was greater at more hyperpolarized potentials with a maximal blockade of 90% at −20 mV, which decreased to 77% at 0 mV. (*D*) Dose–response relationships for C6 inhibition of hHv1 studied at +40 mV (black), +20 mV (dark gray), and 0 mV (light gray). The inhibition constant *K*_i_ of C6 for hHv1 channels at +40 mV, +20 mV, and 0 mV were estimated from the fit to Eq. **1** to be 30.9 ± 3.4 nM (with a Hill coefficient of *h* = 0.48 ± 0.04), 4.9 ± 0.8 nM (*h* = 0.69 ± 0.05), and 1.5 ± 0.2 nM (*h* = 1.22 ± 0.17), respectively (Table 1). Some error bars are smaller than symbols. (*E*) The time course for block and unblock of hHv1 on acute application (red bar) and washout of 250 nM C6 recorded at +40 mV (black), +20 mV (dark gray), and 0 mV (light gray). The association rate constants (*k*_on_) and dissociation rate constants (*k*_off_) were approximated from the kinetics of block and unblock using Eqs. **3** and **4**, relationships derived for bimolecular binding reactions, because the kinetics were well-fitted with single exponentials (Table 1). (*F*) Effect of voltage on C6 blocking kinetics. The *k*_on_ was insensitive to voltage, whereas *k*_off_ and *K*_i_ were responsive. Blocking parameters, *k*_on_ (▾), *k*_off_ (▴), and *K*_i_ (●), were normalized to its value at 0 mV. *K*_i_, *k*_on_, and *k*_off_ were determined as described in *D* and *E*. Error bars are smaller than symbols. (*G*) Conductance–voltage relationships (G–V) for hHv1 (▪), hHv1-R205N (▴), and hHv1-R211S (▾). Curves are fitted to the Boltzmann equation (*Materials and Methods*). The V_1/2_ of *G*–*V* of channels are reported in *SI Appendix*, Table S1. (*H*) Dose–response relationship for C6 inhibition of hHv1-R205N studied at +40 mV. (*H*, *Inset*) Representative current traces with 1 μM C6 (red) or without C6 (black). (*I*) Dose–response relationship for C6 inhibition of hHv1-R211S studied at +40 mV. (*I, Inset*) Representative current traces with 1 μM C6 (red) or without C6 (black).

Before the development of C6, known hHv1 inhibitors were pharmacologically promiscuous or of low affinity (8–10). Synthesized C6 inhibits hHv1 by binding with positive cooperatively to the external channel residues linking the S3 and S4 transmembrane spans (S3–S4 loop), one peptide on each loop, and holds the voltage sensor in a conformation that favors channel closure (Fig. 1*A*) (6). As a result, the channel passes fewer H^+^ ions because more positive voltages are required to open the pores. C6 does not inhibit *Ciona intestinalis* Hv1 (CiHv1) and other voltage-gated K^+^, Na^+^, and Ca^2+^ channels that we tested (6). Single-molecule total internal reflection fluorescent (smTIRF) microscopy was used to show that C6 binds to hHv1 expressed in HEK293T cells with a dissociation constant (*K*_d_) of 0.75 nM at resting membrane potential (−49 mV) (6). C6 affinity for hHv1 decreases with depolarization, achieving just 50% inhibition with 1 μM C6 at +40 mV with a partial block inhibition constant (*K*_i_) of 31 nM (6).

Here, we demonstrate that, as with natural gating modifier toxins, C6 dissociation is accelerated by depolarization (11, 12). As expected, mutations in the S4 of hHv1 that favor the open-channel state show a lower affinity for C6, while mutants that favor channel closure enhance C6 inhibition. Using point mutations, we identified five residues in the hHv1 S3–S4 loop that alter the free energy of blockade (ΔΔG) by more than 2 kcal/mol and that we therefore characterize as important for C6 binding. Taking advantage of a membrane-tethered toxin method (13), we scanned 35 noncysteine residues in tethered C6 (T-C6) and identified 7 that also significantly decrease affinity. We also show that C6 partitions most readily into lipid membranes that contain negatively charged phospholipids. Based on these screening results, molecular dynamics (MD) simulations were carried out to predict energetically important residue–residue interactions in the binding of C6 to hHv1 and the potential role of the lipid bilayer in inhibition. Three predicted C6–hHv1 interaction pairs were confirmed by thermodynamic mutant-cycle analysis, supporting the presented structural model of the complex. This model underpins a gating-modification mechanism through which binding of C6 holds the S4 helix of hHv1 in the closed “down” conformation. hHv1 operates differently than well-studied voltage-gated channels like those for K^+^, Na^+^, and Ca^2+^ ions that have one central pore and four peripheral voltage sensors. Our findings assess the operation and ICK toxin inhibition of H^+^ channels that are dimeric, with two pores, one within each voltage sensor.

Natural bivalent toxin peptides, such as DkTx and Hi1a, isolated from spider venoms have been shown to modify the function of TRPV1 and ASIC1a channels, respectively. The natural toxins act with superior affinity due to their extremely slow dissociation rate (14, 15) and comprise two nonidentical ICK motifs that target two adjacent binding epitopes in the channel (16). Guided by the C6–hHv1 complex model, we constructed a homobivalent C6 (C6_2_) linking two C6 peptides via a 10-residue linker and show that it fully inhibits hHv1 at +40 mV with a *K*_i_ of 630 pM. The importance of blocking the channel at positive potentials is clear when considering neutrophils that depolarize during the inflammatory respiratory burst to +58 mV (17), a voltage where monomeric C6 has low affinity. C6_2_ offers a powerful tool for basic studies of hHv1 and serves with the state-dependent blocker C6 as a lead for studying treatment of diseases where the channel contributes to pathology, including pulmonary damage by leukocytes in pneumonia and acute respiratory distress syndrome (an inflammatory lung disease that is lethal in 40% of patients) (18), ischemic stroke (19), cancer (20), and both neuropathic and inflammatory pain (21, 22).

## Results

### State-Dependent Inhibition of hHv1 by C6.

We previously reported the construction of an ICK scaffold phage-display library with over 1 million variants (*SI Appendix*, *Materials and Methods*) (6). The ICK scaffold is widely seen in venom toxins that bind to the VSDs of a variety of voltage-gated channels and modify the movement of the voltage sensors. Toxin variants possessing the ICK scaffold are stabilized by six cysteines that form three intramolecular disulfide bonds (*SI Appendix*, Fig. S1) and are expressed on the phage surface via encoding into coat protein pIII, allowing for screening. C6 was isolated by sorting the library on purified hHv1 channels (*SI Appendix*, *Materials and Methods*). Stably bound phage were enriched by washing to remove weak and nonspecific interactions, followed by elution, amplification, and rebinding through five rounds of panning. The most enriched phagemids expressed C6 and were increased ∼65,000-fold from starting abundance. Subsequent characterization using smTIRF microscopy showed that the C6 peptide binds to hHv1 expressed in HEK293T cells with a *K*_d_ of 0.75 nM at −49 mV (a potential favoring the channel closed state), whereas the *K*_i_ is attenuated to 31 nM at +40 mV, as determined by using whole-cell patch clamp to study the block of current passed by the open channels (6). Arguing for positive cooperativity of C6 binding at resting membrane potential, such that binding of the first C6 on the dimeric channel facilitates binding of a second C6 on the other subunit, we found that the dose–binding curve was well-fitted using the Hill equation with a coefficient of 1.5 and, further, a fit using the Monod–Wyman–Changeux relationship estimated the first C6 to bind with ∼12-fold lower affinity than the second C6 at the two allosterically coupled sites (6).

To explore the mechanism of voltage-dependent inhibition of C6, hHv1 channels were expressed in HEK293T cells and studied by whole-cell patch clamp with a 10-fold proton gradient (pH_i_ = 6.5 and pH_o_ = 7.5), as before (6). Here, by assessing C6 blocking parameters, we show that C6 affinity for hHv1 decreases on membrane depolarization, largely due to acceleration in the C6 dissociation rate. This is recapitulated by point mutations of “gating charge” residues in the hHv1 S4 helix that respond to voltage to mediate channel opening.

At +40 mV, 250 nM C6 decreased hHv1-mediated outward H^+^ currents by ∼45% (Fig. 1*B*). C6 inhibition was greater at less depolarized potentials, showing a fraction of blocked current of ∼58% at +20 mV and ∼77% at 0 mV (Fig. 1*C*). Fitting the dose–response curve with a Hill equation (Eq. **1**) at +40 mV gave a *K*_i_ for C6 of 31 ± 3 nM with a coefficient of 0.48 ± 0.04 for partial hHv1 blockade (Fig. 1*D* and Table 1), where F_un_ is the fraction of unblocked current at equilibrium, [C6] is the effective C6 concentration, and h is the Hill coefficient, as before (6).

[1] $${\text{F}}_{\text{un}}={\left(1+{\left(\left[\text{C6}\right]\text{/}K_{\text{i}}\right)}^{\text{h}}\right)}^{-1}.$$

The dose–response for the C6 block of hHv1 at +20 mV and 0 mV plotted similarly (Fig. 1*D*) offered *K*_i_ values of 4.9 ± 0.8 nM, with a Hill coefficient of 0.69 ± 0.05, and 1.5 ± 0.2 nM, with a Hill coefficient of 1.22 ± 0.17, respectively (Table 1).

**Table 1.** C6 and C6_2_ blockade of hHv1 channels

Peptide	Channel	Voltage (mV)	*K*_i_ (nM)	Hill coefficient	*k*_on_ (M^-1^⋅s^−1^)	*k*_off_ (s^−1^)
C6	hHv1	0	1.5 ± 0.2	1.22 ± 0.17	5 × 10^5^ ± 1 × 10^5^	0.0015 ± 0.0001
C6	hHv1	+20	4.9 ± 0.8	0.69 ± 0.05	4 × 10^5^ ± 1 × 10^5^	0.0043 ± 0.0002
C6	hHv1	+40	31 ± 3	0.48 ± 0.04	3 × 10^5^ ± 0.8 × 10^5^	0.022 ± 0.001
C6_2_	hHv1	0	0.041 ± 0.003	1.07 ± 0.03	1 × 10^6^ ± 0.2 × 10^6^	5.7 × 10^−5^ ± 0.9 × 10^−5^
C6_2_	hHv1	+40	0.63 ± 0.05	1.01 ± 0.03	9 × 10^5^ ± 1 × 10^5^	7 × 10^−4^ ±1 × 10^−4^
C6	hHv1-R205N	+40	161 ± 19	0.5 ± 0.1	3 × 10^5^ ± 0.5 × 10^5^	0.19 ± 0.03
C6	hHv1-R211S	+40	0.6 ± 0.1	0.4 ± 0.1	5 × 10^5^ ± 1 × 10^5^	0.0008 ± 0.0001

hHv1, hHv1-R205N, and hHv1-R211S channels were expressed in HEK293T cells and studied by whole-cell patch clamp. The blockade parameters were assessed by using a holding voltage of −60 mV and 1.5-s test pulses at test voltages, as described in Fig. 1. The *K*_i_ and Hill coefficients of peptides for hHv1 channels were estimated from a fit of the dose–response to Eq. **1**. The *k*_on_ and *k*_off_ were determined from fits of the kinetics of block and unblock of hHv1 on acute application and washout of 250 nM C6 or 10 nM C6_2_ and approximated by using Eqs. **3** and **4**, relationships derived for bimolecular binding reactions. Values are mean ± SEM; *n* = 3 to 6 cells for each condition.

The *K*_i_ for toxin block can also be estimated by the ratio of the first-order dissociation rate constant (*k*_off_) and the second-order association rate constant (*k*_on_), according to Eq. **2**.

[2] $$K_{\text{i}}=k_{\text{off}}\text{/}k_{\text{on}}.$$

Furthermore, *k*_on_ and *k*_off_ are related to the association constant (*τ*_on_) and dissociation constant (*τ*_off_) derived from single-exponential fits of the time courses for toxin block and unblock by Eqs. **3** and **4**.

[3] $$\tau_{\text{on}}={\left(k_{\text{on}}\left[\text{Tx}\right]+k_{\text{off}}\right)}^{-1},$$

[4] $$\tau_{\text{off}}={\left(k_{\text{off}}\right)}^{-1}.$$

Thus, the time course of wash-in and washout of 250 nM C6 can be fitted with single exponentials, allowing approximation of the time course of inhibition, *k*_on_ = 3 × 10^5^ ± 0.8 × 10^5^ M^−1^⋅s^−1^, and recovery, *k*_off_ = 0.022 ± 0.001 s^−1^ at +40 mV (Fig. 1*E*) (6). Comparing the kinetics of C6 wash-off at various potentials made it readily apparent that toxin dissociation was strongly dependent on voltage (Fig. 1*E*), showing an ∼15-fold slower dissociation at 0 mV than +40 mV, whereas the C6 association rate was slowed only ∼1.6-fold at + 40 mV (Fig. 1*F* and Table 1). Of note, the positive cooperativity of C6 binding to the second subunit observed at −49 mV showed a Hill coefficient of 1.5 (6), which we demonstrate here by studying current-inhibition decreases with depolarization from 1.22 at 0 mV to 0.48 at +40 mV, the last value suggestive of negative cooperativity (Table 1). Thus, the simplifying utility of Eqs. **3** and **4**, derived for bimolecular binding reactions, and the use of single-exponential fits represent a practical strategy to approximate parameters of C6 binding to the two sites in hHv1 that are allosterically coupled differently at various voltages.

The S4 helix of hHv1 contains three conserved Arg residues (R205, R209, and R211), which confer the channel with sensitivity to voltage (3). These conserved Arg residues form salt bridges with acidic residues in the S1–S3 helices during state transitions, similar to conventional voltage-gated channels and enzymes (23–26). We neutralized two Arg residues in the hHv1 S4 and observed conductance–voltage (*G*–*V*) relationship shifts consistent with the hypothesis that R205 helps maintain a closed state of hHv1, while R211 contributes to stabilizing an open state (1, 27). Thus, the hHv1-R205N mutation shifted *V*_1/2_ by approximately −57 mV, favoring channel opening, while the hHv1-R211S mutation shifted the *V*_1/2_ by approximately +33 mV, favoring the closed state (Fig. 1*G* and *SI Appendix*, Table S1). To investigate state-dependent inhibition by C6, its dose-dependence was studied at +40 mV, and the results fit with Eq. **1**. As predicted, hHv1-R205N showed a *K*_i_ for C6 blockade of 161 ± 19 nM, with a Hill coefficient of 0.5 ± 0.1, whereas hHv1-R211S showed a *K*_i_ = 0.6 ± 0.1 nM, with a Hill coefficient of 0.4 ± 0.1 (Fig. 1 *H* and *I* and Table 1). Compared to wild-type (WT) hHv1, C6 blocking kinetics of R211S at +40 was slowed ∼28-fold (Table 1), and it was accelerated ∼9-fold by the R205N mutation (Table 1). This is consistent with the idea that C6 dissociates more rapidly from the open-channel state, leading to higher affinity in the closed state.

### Mapping the Residues in the hHv1 S3–S4 Loop Critical for C6 Binding.

**Fig. 2.**
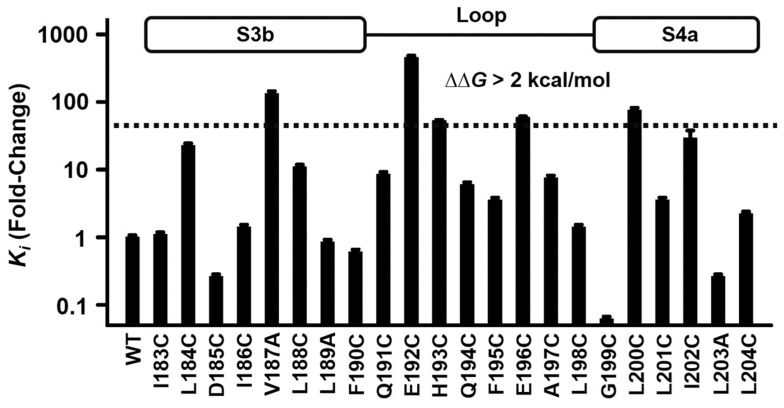
Effects of mutations in the S3–S4 loop of hHv1 on C6 affinity. hHv1 and point mutant channels were expressed in HEK293T cells, and the *K*_i_ for C6 was determined by whole-cell patch clamp, as in Fig. 1. Because some of the point mutants show a right shift in the G–V relationship (*SI Appendix*, Table S1), they pass almost no H^+^ current at 0 mV; therefore, all mutants were studied at +40 mV for comparison. The *K*_i_ of 14 mutants were determined by studying the response to varying doses of C6 (8 are shown in this figure), and based on those findings, the *K*_i_ for the other 14 mutants were estimated by using Eq. **1** and assuming the same Hill coefficient as determined for the WT channel (*SI Appendix*, Table S1). Changes of free energy of C6 blockade (ΔΔG) were calculated by using Eqs. **5** and **6**. Values are normalized to the *K*_i_ of C6 for WT hHv1. Values are mean ± SEM; *n* = 3 to 6 cells for each construct.

We showed that C6 binds to the external S3–S4 loop of hHv1 by “transplanting” the hHv1 loop region (I183 to L204) into the C6-insensitive channel CiHv1, conferring the chimeric channel with toxin sensitivity similar to that of WT hHv1 (6). To explore the role of individual S3–S4 loop residues in C6 binding, 22 loop residues (I183 to L204) were individually mutated, and C6 blockade of the mutant hHv1 channels was studied at +40 mV using whole-cell patch clamp (Fig. 2). Of the 22 mutant channels, hHv1-V187C, hHv1-E192C, hHv1-H193C, hHv1-E196C, and hHv1-L200C showed changes in the ΔΔG of C6 blockade of more than 2 kcal/mol (*K*_i mutation_/*K*_i WT_ > 43-fold; Fig. 2 and *SI Appendix*, Table S1), according to Eqs. **5** and **6**, where R is the gas constant and T is the temperature in degrees Kelvin.

[5] $$K_{\text{i}\;\text{mutation}}\text{/}K_{\text{i}\;\text{WT}}={\left[\left(1–{\text{F}}_{\text{un}\;\text{WT}}\right){\;\text{F}}_{\text{un}\;\text{mutation}}/\left(1–{\text{F}}_{\text{un}\;\text{mutation}}\right){\;\text{F}}_{\text{un}\;\text{WT}}\right]}^{1/\text{h}},$$

[6] $$\Delta \Delta \text{G}=\text{RT}\;\text{ln}\left(K_{\text{i}\;\text{mutation}}\text{/}K_{\text{i}\;\text{WT}}\right).$$

By consensus, a value of 2 kcal/mol is used as the cutoff for ΔΔG to identify residues most likely to be engaged in statistically relevant energetic interactions at protein–protein interaction interfaces (28, 29). Thus, the observed large free-energy changes in C6 binding suggest that these five channel residues may mediate direct interaction with C6. We also found three mutations (hHv1-D185C, hHv1-G199C, and hHv1-L203C) that enhanced C6 inhibition more than fourfold (Fig. 2 and *SI Appendix*, Table S1). Among them, hHv1-G199C is noteworthy for its ∼16-fold improvement in the C6 *K*_i_ (1.9 ± 0.2 nM) compared to WT hHv1, with no change in the *G*–*V* relationship (*SI Appendix*, Table S1).

### Identification of Critical Toxin Residues Using T-C6 Variants.

**Fig. 3.**
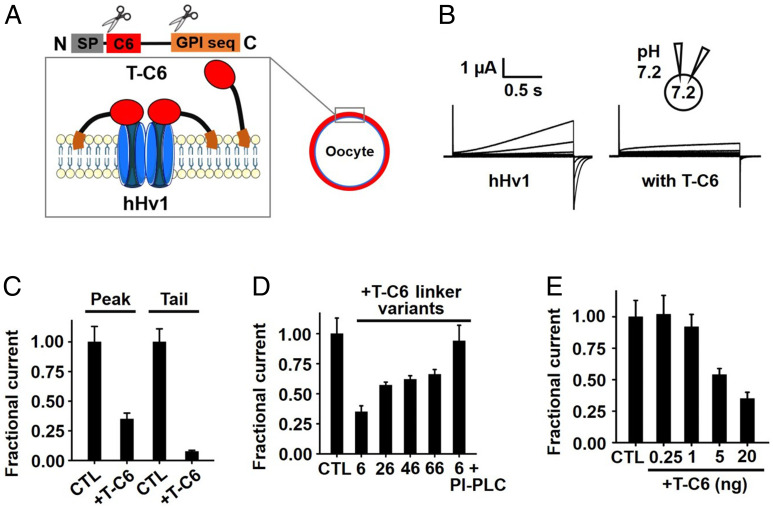
T-C6 blocks hHv1 channels. hHv1 expressed in *Xenopus* oocytes was studied by TEVC to assess T-C6 inhibition at equilibrium from a holding voltage of −60 mV with 1.5-s test pulses and 10-s interpulse intervals, with symmetric pH_o_ and pH_i_ = 7.2 maintained by injection of 50 nL of 1 M Hepes into oocytes (*Materials and Methods*). Values are mean ± SEM; *n* = 12 cells for each condition. (*A*) T-C6 was constructed as a chimeric fusion protein with an N-terminal secretory signal sequence (SP; gray), the C6 sequence (red), a hydrophilic flexible linker (six residues), and a C-terminal GPI membrane anchor targeting sequence (orange) (*SI Appendix*, Fig. S2). The colors in the schematic mark T-C6 components. (*B*) Representative current traces (steps from −60 to +80 mV) for hHv1 channels without (CTL) or with 20 ng of T-C6 cRNA coinjection. (*C*) hHv1 currents at +80 mV (Peak) and -60 mV (Tail) with T-C6 (20 ng of cRNA) normalized to the unblocked condition (CTL). (*D*) T-C6 linker variants with hydrophilic flexible linkers ranging from 6 to 66 residues show different extent of inhibition to hHv1. The average proton currents at +80 mV with T-C6 linker variants (20 ng of cRNA) were normalized to the unblocked condition (CTL). PI-PLC treatment reverses blockade by T-C6 (six-residue linker). (*E*) Concentration-response for T-C6 inhibition of hHv1 currents at +80 mV normalized to the unblocked condition (CTL).

The in vitro synthesis and folding of venom-toxin peptides can be technically challenging, costly, and time-consuming. To facilitate characterization of C6 by mutational screening, we employed a strategy whereby C6, or its variants, were expressed from a gene in tethered form on the extracellular surface of cells via a glycosyl phosphatidyl inositol (GPI)-anchored membrane-embedded leash, as we have before with other toxins (13). The construct included a signal peptide sequence, C6 or its variants, and a flexible linker, followed by a GPI targeting sequence (Fig. 3*A*, *SI Appendix*, Fig. S2*A*, and *Materials and Methods*). This permitted study of the inhibition of hHv1 by T-C6 and variants in *Xenopus* oocytes using two-electrode voltage clamp (TEVC) (13, 30). Complementary RNA (cRNA) for T-C6 was synthesized in vitro and coinjected with cRNA encoding hHv1 into oocytes. The result was T-C6 expressed on the extracellular leaflet of the oocyte membrane via the GPI anchor after natural cleavage of the signal peptide, as well as hHv1 channels (Fig. 3 *A* and *B*). When hHv1 currents were measured 3 d after injecting 10 ng of channel cRNA and 20 ng of T-C6 cRNA, blockade was ∼65% of the outward H^+^ current at +80 mV and ∼92% of the tail current at −60 mV (Fig. 3 *B* and *C*); this was the equivalent of adding 30 μM C6 peptide to the bath solution. The impact of the length of linker between C6 and the GPI anchor was evaluated by using constructs with 6, 26, 46, and 66 intervening residues. All constructs blocked hHv1 effectively, with the six-residue linker offering the greatest inhibition (Fig. 3*D*), and this was used subsequently for scanning mutagenesis. Demonstrating the reversibility of T-C6 inhibition, treatment with phosphatidylinositol-specific phospholipase C (PI-PLC) that cleaves GPI-anchored proteins from the oocyte surface restored outward currents to control levels (Fig. 3*D*). When T-C6 cRNA was injected in amounts from 1 ng to 20 ng, outward H^+^ current inhibition increased from 8 to 65% (Fig. 3*E*).

**Fig. 4.**
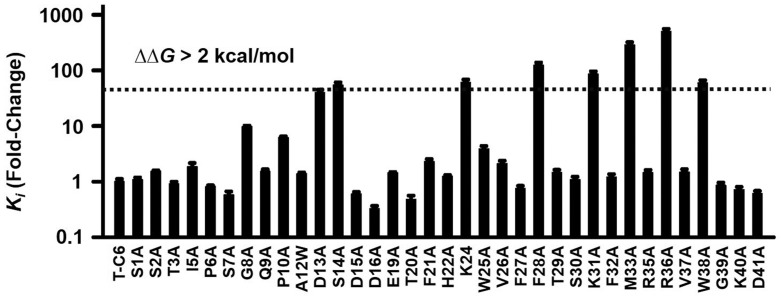
T-C6 screening mutagenesis. hHv1 was expressed in oocytes and inhibition by T-C6, and variants were studied by TEVC after coinjection of 20 ng of the cRNAs. hHv1 currents at +80 mV with T-C6 variants were measured as in Fig. 3. A pulse voltage of +80 mV was used for variant screening to generate sufficiently large H^+^ currents in oocytes under symmetrical conditions (pH_o_ = pH_i_ = 7.2). The change in *K*_i_ for the mutants was estimated relative to WT using Eq. **5** and assuming the same Hill coefficient as determined for the WT channel. The changes of free energy of C6 blockade (ΔΔG) were calculated by using Eqs. **5** and **6**. Values are mean ± SEM; *n* = 12 cells for each variant.

C6 has 41 residues with three disulfide bonds that maintain the ICK toxin scaffold. To identify the C6 residues that might mediate direct interaction with the S3–S4 loop of hHv1 (or membrane lipids around the channels), we generated T-C6 point mutations at the 35 positions that were not Cys (Fig. 4). Injection of 20 ng of WT T-C6 cRNA inhibited 65% of hHv1 outward current at +80 mV, and seven of the T-C6 variants inhibited less than 23%, corresponding to a change in ΔΔG of more than 2 kcal/mol, according to Eqs. **5** and **6** (Fig. 4). The most dramatic effect on inhibition was seen with T-C6–R36A, which decreased the *K*_i_ by ∼500-fold. Other mutations with high impact were S14A, K24A, F28A, K31A, M33A, and W38A (Fig. 4).

To confirm that the effects of mutations were not due to changes in surface expression, we constructed the same mutations in T-C6 with the 46-residue linker (T-C6_46_; *SI Appendix*, Fig. S2*B*), which carries a c-Myc epitope, allowing comparison of the surface levels of T-C6_46_ and variants by enzyme-linked immunosorbent assay (ELISA), as we have described (13). Injection of 20 ng of T-C6_46_ cRNA inhibited 39% of hHv1 outward current (Fig. 3*D*); the seven critical mutations of T-C6_46_ produced changes in *K*_i_ like the same mutations in T-C6 (*SI Appendix*, Fig. S3 and Table S2), and none showed a significant change in surface expression by ELISA, ruling out altered expression as the cause for lowered blockade (*SI Appendix*, Fig. S3).

### C6 Partitioning into Cell Membranes.

**Fig. 5.**
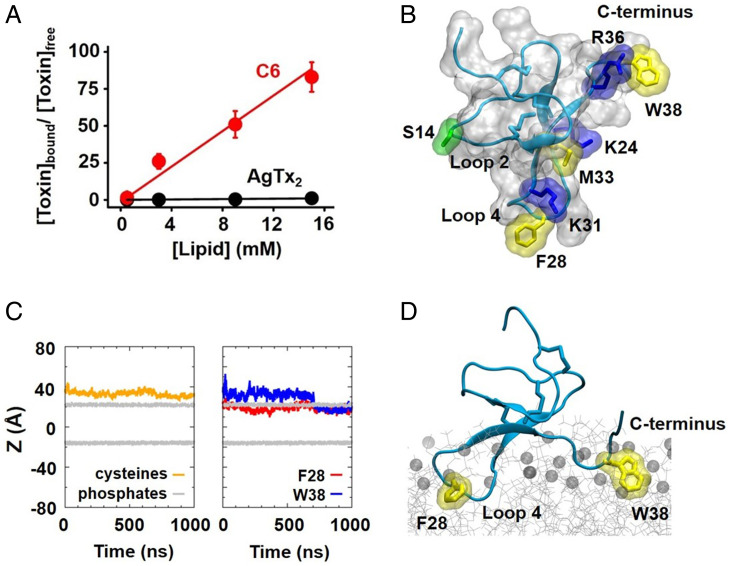
Partitioning of C6 into lipid membranes. A depletion assay (*Materials and Methods*) was used to determine the partitioning of C6 into lipid membranes. LUVs were prepared with POPC:POPG (3:1) and incubated with C6 solution. C6 peptide remaining in the aqueous phase was quantitated by HPLC (*SI Appendix*, Fig. S4). The C6 structure was modeled based on the NMR structure of Mu-agatoxin-Aa1a (PDB ID code: 1EIT) using the MODELER program, and the interaction between C6 and POPC:POPG (3:1) lipids was simulated by using the NAMD program, as described in *Materials and Methods*. (*A*) Ratio of bound/free C6 (red) or AgTx2 (black) plotted as a function of LUVs concentration. Values are mean ± SEM; *n* = 3 for each condition. Some error bars are smaller than symbols. (*B*) Surface and ribbon representations of the homology model of C6. The heavy side-chain and Cα atoms of key residues derived from screening mutagenesis (Fig. 4) are highlighted in stick-and-surface representation. Three disulfide bonds (C4–C18, C11–C23, and C17–C34) that demonstrate a classic ICK scaffold are highlighted in stick representation. Basic, polar, and hydrophobic residues are colored blue, green, and yellow, respectively. (*C*) Time series of the z coordinate of the center-of-mass of Cα atoms of the six cysteines (orange line) of C6, phosphate atoms in the upper and inner lipid leaflets (gray lines), and the heavy side-chain atoms of F28 (red line) and W38 (blue line) of C6, respectively, in the MD simulations system with a POPC:POPG lipid bilayer. (*D*) Snapshot of C6 partitioning into a POPC:POPG lipid bilayer using its hydrophobic motifs in the MD simulations. Phosphate atoms of lipids are shown in sphere representation (only part of the lipids are shown for clarity). The two hydrophobic residues of C6, F28 and W38 (yellow), inserting into the lipid bilayer are shown in stick-and-surface representation. Three disulfide bonds are shown in stick representation (cyan).

ICK toxins like HaTx and ProTx2 from spiders inhibit voltage-gated channels by first partitioning into the plasma membrane, followed by direct interaction with VSDs (31, 32). We studied the partitioning of C6 into large unilamellar vesicles (LUVs) composed of a 3:1 mixture of zwitterionic 1-palmitoyl-2-oleoyl-*sn*-glycero-3-phosphocholine (POPC) and anionic 1-palmitoyl-2-oleoyl-*sn*-glycero-3-phosphoglycerol (POPG) phospholipids using a toxin-depletion assay (33). Varying amounts of LUVs were incubated with C6 in solution, and liposome-bound C6 was separated by ultracentrifugation. C6 remaining in the aqueous phase was quantified by using reverse-phase, high-performance liquid chromatography (HPLC). Roughly 50% of C6 peptide partitioned into 0.5 mM LUVs (Fig. 5*A* and *SI Appendix*, Fig S4*A*). This increased to 99% when the LUVs’ concentration was raised to 15 mM (Fig. 5*A*). As a control, AgTx2, a scorpion toxin that binds in the external pore region of K^+^ channels (34), did not partition into LUVs (Fig. 5*A*). The mole fraction partition coefficient (*K*_p_) of C6 estimated from the titration data was 3.2 × 10^5^ ± 0.4 × 10^5^ using Eq. **7**, where the fraction of C6 partitioned into the membrane (F_p_) is equal to ([C6_total_] − [C6_free_])/[C6_total_], [L] is the LUVs’ concentration, and [W] is the molar concentration of water (55.3 mol/L).

[7] $$K_{\text{p}}=\left[{\text{F}}_{\text{p}}\text{/}\left(1-{\text{F}}_{\text{p}}\right)\right]\left[\text{W}\right]/\left[\text{L}\right].$$

The *K*_p_ of C6 is comparable to that reported for HaTx (5.3 × 10^5^) (31), suggesting that C6, like other natural ICK toxins, interacts with membrane lipids (31, 32). To confirm that C6 partitions into native membranes, we examined the ability of intact *Xenopus* oocytes and HEK293T cells to deplete C6 from physiological solutions (*Materials and Methods*). When cells are agitated gently in a solution containing C6, we observed depletion of 34% and 67% of C6 from the aqueous phase by oocytes and HEK293T cells, respectively (*SI Appendix*, Fig. S4*B*). In contrast, others have shown that less than 1% of AgTx_2_ toxin peptide was depleted by the same number of oocytes (31).

### A Homology Model of C6 and its Interaction with Lipids.

Mu-agatoxin-Aa1a is a spider ICK toxin that activates insect voltage-gated Na^+^ channels (35) and shows 40% identity to the C6 peptide sequence (*SI Appendix*, Fig. S5 *A–C*). The NMR structure of Mu-agatoxin-Aa1a (Protein Data Bank [PDB] ID code 1EIT) was chosen as a template to generate a C6 homology model (*Materials and Methods*). Our C6 model employs a standard ICK scaffold with four solvent-accessible loops protruding from a disulfide-bonded globular core (Fig. 5*B* and *SI Appendix*, Fig. S5*A*). C6 has two hydrophobic patches on its surface, W25–F28 in loop 4 and V37–W38 at the C terminus (Fig. 5*B* and *SI Appendix*, Fig. S5*A*). These six solvent-exposed hydrophobic residues form two prime motifs for membrane partitioning. Five positively charged basic residues (K24, K31, R35, R36, and K40) were arranged around the edge of the hydrophobic motifs, while four out of the five negatively charged acidic residues (D13, D15, D16, and E19, but not D41) clustered on the other side of the structure (*SI Appendix*, Fig. S5*G*). We mapped the mutagenesis results onto the C6 homology-modeling structure to show the position of the C6 residues critical for blocking hHv1 (Fig. 5*B*).

One face of C6 stands out as containing six out of the seven critical residues (K24, F28, K31, M33, R36, and W38), where mutations to Ala increased *K*_i_ values by >43-fold (ΔΔG > 2 kcal/mol; Fig. 4). These residues are arrayed in a half concentric ring that we anticipate forms intimate interactions with the VSD in hHv1 (Fig. 5*B*). In contrast, residue S14 in loop 2 located on the opposite side of C6 most likely contributes to toxin conformation stability (36) or lipid interaction (16).

To investigate the interaction of C6 and lipids, we constructed an all-atom system of C6 and a fully hydrated lipid bilayer of POPC:POPG (3:1), as used in the toxin-depletion assay (Fig. 5*A*), with the C6 placed initially in the extracellular solution (*Materials and Methods*). During 1-µs MD simulations, C6 spontaneously partitioned into POPC:POPG lipids with the bulky side chain of F28 initially and W38 later, anchoring deep into the hydrophobic core of the lipid bilayer (Fig. 5 *C* and *D*). In contrast, C6 was not able to partition into a bilayer of pure POPC lipids in our MD simulations (*SI Appendix*, Fig. S5 *H* and *I*). Consistent with the simulation, the *K*_p_ of C6 for binding to POPC liposomes was 3,000-fold less than with POPC:POPG (3:1) (1.1 × 10^2^ ± 0.1 × 10^2^) (*SI Appendix*, Fig. S5*J*). Thus, C6 partitions into membranes containing anionic lipids like other natural VSD toxin modifiers (31, 37). Analysis of the simulation trajectories showed that electrostatic interactions between C6 basic residues and anionic lipids facilitated the dwell time of the toxin on the membrane surface. This is promoted by the formation of extensive salt bridges between the basic residue side chains and the phosphate and ester oxygen atoms of lipids (*SI Appendix*, Fig. S5 *G* and *I*), leading to the insertion of bulky hydrophobic residues into the hydrophobic core of lipids (Fig. 5 *C* and *D*).

### A Proposed Model of C6 Binding to the hHv1 Closed State.

**Fig. 6.**
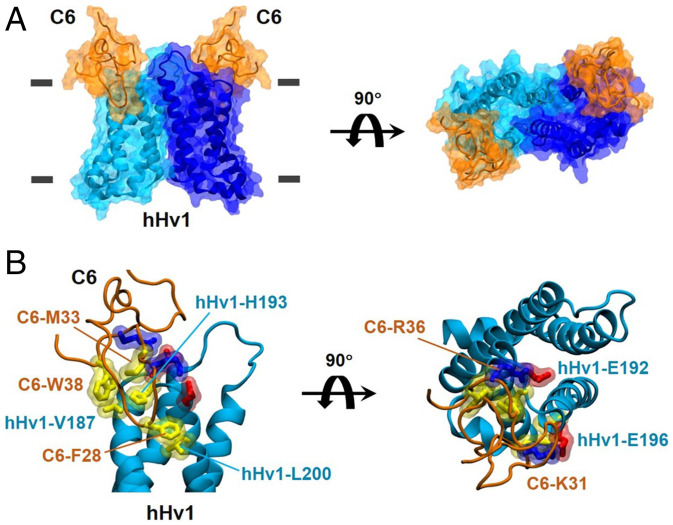
C6–hHv1 structural model from MD simulations. The homology model of C6 and our down-state dimeric hHv1 model (23) were used to build a structural model of the C6–hHv1 complex using MD simulations (*Materials and Methods*) with knowledge of the orientation of C6 anchoring into a POPC:POPG lipid bilayer (Fig. 5) and the important toxin-channel interaction residues identified by electrophysiological mutant scans (Figs. 2 and 4). (*A*) The C6–hHv1 complex in two orientations showing that two C6 molecules (orange) bind to the two subunits (cyan and blue) of an intact dimeric hHv1 channel. The transmembrane helices S1 and S4 form the dimer interface. The horizontal gray lines indicate the position of the membrane boundary. (*B*) Close-up view of the interaction interface between one C6 and one hHv1 subunit in the structural model. F28, M33, and W38 (yellow side chains) of C6 form hydrophobic interactions with L200, H193, and V187 (yellow side chains) of hHv1, respectively. K31 and R36 (blue side chains) of C6 form salt bridges with E196 and E192 (red side chains) of hHv1, respectively.

Based on our knowledge of the orientation of C6 partitioning into lipids (Fig. 5*D*) and the toxin and channel residues identified as critical for binding by patch clamp (Fig. 4), a structural model of the C6–hHv1 complex was constructed and refined by using MD simulations (Fig. 2). As the starting structures for the complex modeling, the transmembrane regions (residues G90 to I218) of the homology model of dimeric hHv1 in its electron paramagnetic resonance-validated closed state (*SI Appendix*, Fig. S6 and *Materials and Methods*) (23) and the homology model of C6 (Fig. 5*B*) were used. Two C6 molecules were placed in the extracellular solution with the loop-4 region facing the membrane and the S3–S4 loop of each subunit of the channel. Stepwise distance restraints were applied between C6 and lipids, and C6 and hHv1, to move C6 toward hHv1 (*Materials and Methods*). The final C6–hHv1 complex model (Fig. 6*A*) displays specific interactions consistent with the experimental observations from scanning mutagenesis of the channel and C6. In particular, the model exhibits electrostatic interaction between hHv1-E192 and C6-R36, hHv1-E196 and C6-K31; and hydrophobic interactions between hHv1-V187 and C6-W38, hHv1-H193 and C6-M33, and hHv1-L200 and C6-F28 (Fig. 6*B*).

### Validation of the Model by Point Mutations.

Two charge-reversal experiments inspired by our MD simulations further support the model of the C6–hHv1 complex by meeting the expectations for salt bridges between hHv1-E192 and C6-R36 and between hHv1-E196 and C6-K31. When the side chain hHv1-E192 was reversed in charge by mutation to Lys (producing hHv1-E192K), inhibition by T-C6 was reduced ∼52-fold (*SI Appendix*, Fig. S7*A*). When the predicted C6 charge partner, R36, was reversed to Glu (producing T-C6–R36E), toxin inhibition of hHv1-E192K was restored. As a control, two other point mutations, T-C6–K24E and T-C6–K31E, were studied with hHv1-E192K channels, and they did not restore inhibition (*SI Appendix*, Fig. S7*A*). Similarly, T-C6 inhibition was reduced by ∼12-fold with hHv1-E196K channels, and block was restored by the countermutation T-C6–K31E, but the control changes T-C6–K24E and T-C6–R36E did not restore inhibition (*SI Appendix*, Fig. S7*B*). These “side-chain swaps” validate the model position of hHv1-E192 and hHv1-E196 near C6-R36 and C6-K31, respectively.

### Pairwise Interactions Confirmed by Mutation-Cycle Analysis.

We used thermodynamic mutant-cycle analysis to verify pairwise interactions suggested by the C6–hHv1 complex model, an approach brought to the fore by mapping the binding site of scorpion toxins in the pore of the K^+^ channels (38). The interaction between residues on the channel and toxin are identified by making channel mutations (Mut) and toxin variants (Var) and measuring the *K*_i_ for different combinations of modified and unmodified pairs. The coupling energy ΔΔG between Mut and Var can be calculated from the measured *K*_i_ according to Eqs. **8** and **9**. The size of the ΔΔG quantifies the strength of interaction between the Mut and Var.

[8] $$\Omega =(K_{\text{i}\;\text{WT}\;\text{C6},\;\text{WT}\;\text{hHv1}}\times K_{\text{i}\;\text{C6}\;\text{variant},\;\text{hHv1}\;\text{mutation}})\text{/}(K_{\text{i}\;\text{WT}\;\text{C6},\;\text{hHv1}\;\text{mutation}}\times K_{\text{i}\;\text{C6}\;\text{variant},\;\text{WT}\;\text{hHv1}}),$$

[9] ΔΔG=RT lnΩ.

**Fig. 7.**
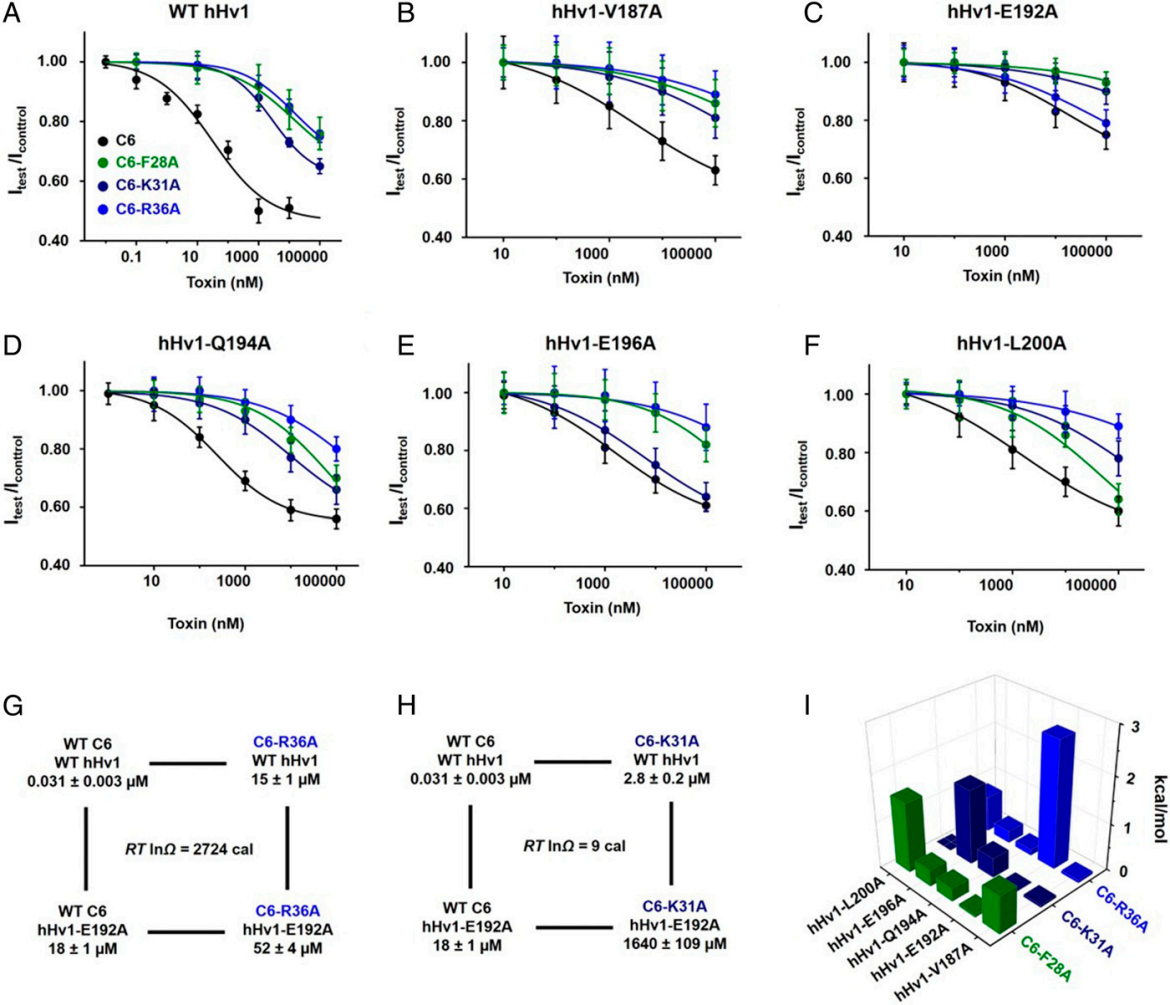
Mutant-cycle analysis validates modeled pairwise interactions between C6 and hHv1. Peptides of C6, C6-F28A, C6-K31A, and C6-R36A were synthesized and tested on hHv1, hHv1-V187A, hHv1-E192A, hHv1-Q194A, hHv1-E196A, and hHv1-L200A in HEK293T cells using whole-cell patch clamp (at +40 mV), as described in Fig. 1. Values are mean ± SEM; *n* = 3 to 6 cells for each condition. (*A–F*) Dose–response curves for the inhibition of WT and mutant channels by C6 and variants. *K*_i_ was determined by fitting the curves to the Hill equation (Eq. **1**). *K*_i_ of all combinations is reported in *SI Appendix*, Table S3. (*G*) A thermodynamic cycle for mutations C6-R36A and hHv1-E192A, showing large coupling energy. Each corner of the cycle represents the *K*_i_ of an indicated toxin-channel combination pair. Ω is defined in Eq. **8**, and the coupling energy is calculated by using Eq. **9**; these are approximations using relationships derived for bimolecular binding reaction. (*H*) In contrast to *G*, a cycle for C6-K31A with hHv1-E192A shows negligible coupling energy (lnΩ ∼ 0), despite the large energetic effect of the C6-K31A mutation alone (Fig. 4). (*I*) Coupling energy values for the indicated combinations of peptide C6 variants and hHv1 mutations. Three toxin-channel pairs, C6-F28 and hHv1-L200 (ΔΔG = 1.5 kcal/mol), C6-K31 and hHv1-E196 (1.6 kcal/mol), and C6-R36 and hHv1-E192 (2.7 kcal/mol), show large coupling energies.

We synthesized peptides for three C6 variants, C6-F28, C6-K31A, and C6-R36A, for the mutant-cycle analysis. On the channel, we investigated mutations of five residues, hHv1-V187A, hHv1-E192A, hHv1-Q194A, hHv1-E196A, and hHv1-L200A, in the S3–S4 loop (Fig. 7). We tested C6 and the three toxin variants on the WT hHv1 and the five channel mutants and determined the dose–response for inhibition of the 24 channel–toxin combinations by whole-cell patch clamp with test steps to +40 mV (Fig. 7 *A–F*). The *K*_i_ estimated by fitting the dose–response curves to Eq. **1** (*SI Appendix*, Table S3) was used to calculate the ΔΔG of coupling for each combination. Fig. 7 *G* and *H* shows two examples of individual mutant-cycle analyses. In Fig. 7*G*, C6-R36A is paired to hHv1-E192A, and the combination produces a ΔΔG of ∼2.7 kcal/mol. In Fig. 7*H*, C6-K31A is paired with hHv1-E192A, and the combination produces a ΔΔG of ∼0.01 kcal/mol. These results support an interaction between hHv1-E192 and C6-R36, but not C6-K31. All values of the ΔΔG are shown in Fig. 7*I*, where three combinations, hHv1-E192 and C6-R36, hHv1-E196 and C6-K31, and hHv1-L200 and C6-F28, show ΔΔG coupling values > 1 kcal/mol, suggesting that these are direct interaction pairs, consistent with the C6–hHv1 model (Fig. 6*B*).

### A Designed Bivalent C6 Inhibits hHv1 Fully at Depolarized Voltages.

**Fig. 8.**
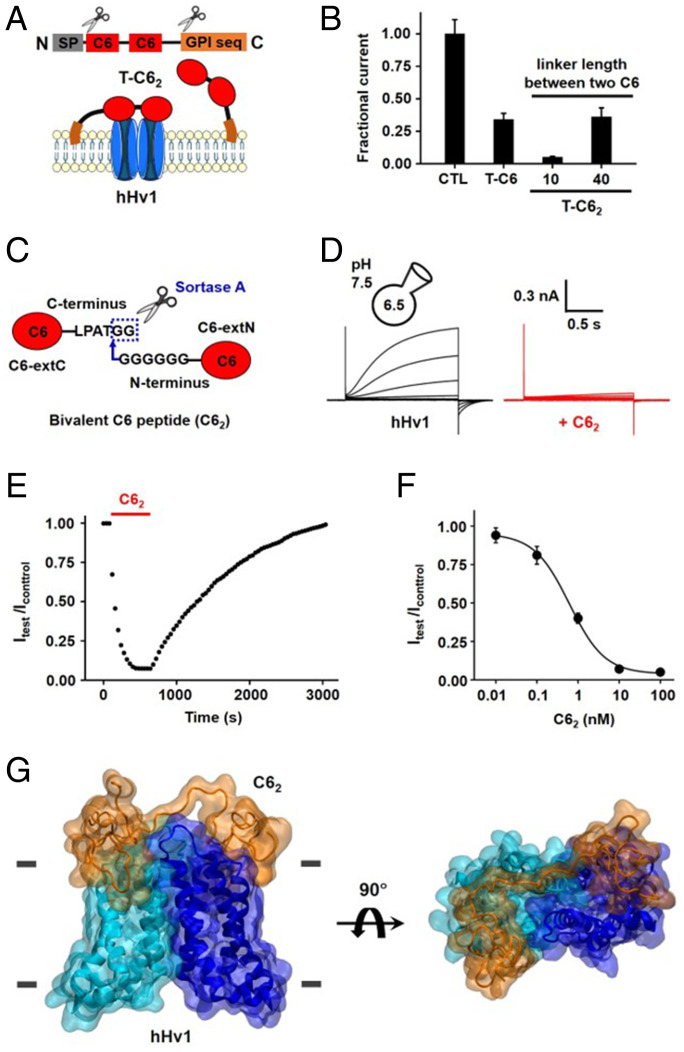
hHv1 Inhibition by C6_2_. Inhibition by T-C6_2_ of hHv1 was studied in oocytes by TEVC, as in Fig. 3 (*n* = 12 cells for each condition), and in HEK293T cells using whole-cell patch clamp, as in Fig. 1 (*n* = 6 cells for each condition). Values are mean ± SEM. (*A*) T-C6_2_ was constructed by using T-C6 and replacing the encoding sequence for C6 with two C6 connected by flexible linkers with either 10 or 40 residues (*Materials and Methods*). (*B*) Inhibition of hHv1 currents in oocytes by T-C6 and T-C6_2_ linker variants (20 ng of cRNA) studied at +80 mV and shown normalized to the unblocked condition (CTL). (*C*) Conjugation of C6 peptides using Sortase A transpeptidase enzyme. C6 peptides with C-terminal (C6-extC) or N-terminal (C6-extN) extensions were synthesized. Sortase A cleaves between Thr and Gly in the recognition sequence at the C terminus of C6-extC and catalyzes the formation of an amide bond with the nucleophilic polyglycine at the N terminus of C6-extN. (*D*) Representative H^+^ current traces for hHv1 channels in HEK293T cells before (*Left*) and in the presence of 10 nM C6_2_ peptide (*Right*) with steps of 20 mV from −60 to +40 mV. The peak current measured at the end of depolarization was used to determine the extent of block. (*E*) The time course for block and unblock of hHv1 in HEK293T cells on acute application (red bar) and washout of 10 nM C6_2_ peptide recorded at +40 mV. (*F*) Dose–response relationships for C6_2_ peptide inhibition of hHv1 in HEK293T cells at +40 mV. The inhibition constant *K*_i_ of C6_2_ for hHv1 channels was estimated from the fit to Eq. **1** to be 631 ± 52 pM with a Hill coefficient of 1.01 ± 0.03. (*G*) The homology model of C6_2_ and the down-state dimeric hHv1 model were used to build a structural model of the C6_2_–hHv1 complex using MD simulations (*Materials and Methods*). The C6_2_–hHv1 complex in two orientations shows two C6 epitopes (orange) connected by a 10-residue peptide linker (LPATGGGGGG) binding simultaneously to the two hHv1 subunits (cyan and blue). The horizontal gray lines indicate the position of the membrane boundary.

In the C6–hHv1 model, the two VSDs are stabilized by two C6 peptides (Fig. 6*A*). The distance between the C terminus of one C6 and the N terminus of the other C6 is ∼30 Å. This inspired the design of a bivalent toxin containing two C6 molecules (C6_2_) that we anticipated would bind to the two hHv1 subunits simultaneously, slowing toxin dissociation, as observed for natural bivalent toxins (16, 39). To examine the idea, we first created two tethered bivalent C6 constructs with either a 10- or 40-residue flexible linker connecting two C6 sequences, T-C6_2(10)_ and T-C6_(40)_, respectively (Fig. 8 *A* and *B* and *Materials and Methods*). Coinjection of 20 ng of cRNA of T-C6_2(10)_ inhibited ∼95% of the outward H^+^ current at +80 mV, far superior to block by the monomer T-C6 (∼65% inhibition) (Fig. 8*B*). In contrast, T-C6_2(40)_ inhibited like WT T-C6, ∼64% of the current.

Bivalent toxin peptides are difficult to synthesize and fold in vitro due to their long and disulfide-rich sequences. Sortase A is a bacterial thiol transpeptidase that can ligate peptides with a C-terminal recognition sequence (LPXTG) to peptides containing an N-terminal polyglycine sequence. Because the reaction conditions do not require a reduced environment and show outstanding efficiency, Sortase A has been used to link toxin peptides (40). Here, we synthesized two C6 peptides, C6-extC and C6-extN, with six additional residues (LPATGG or GGGGGG) at the C terminus or N terminus, respectively, and ligated the two peptides using Sortase A to create a bivalent C6 peptide with a 10-residue connecting linker, LPATGGGGGG (Fig. 8*C* and *Materials and Methods*). C6_2_ produced by the ligation reaction was purified by using HPLC (*SI Appendix*, Fig. S8) and studied on hHv1 channels expressed in HEK293T cells by using whole-cell patch clamp. Application of 10 nM C6_2_ decreased outward H^+^ currents through hHv1 ∼96% at +40 mV (Fig. 8*D*). Acute application and washout of 10 nM C6_2_ gave *k*_on_ = 9 × 10^5^ ± 1 × 10^5^ M^−1^⋅s^−1^ and *k*_off_ = 0.0007 ± 0.0001 s^−1^ at +40 mV (Fig. 8*E*), an on-rate ∼2.6-fold faster and an off-rate ∼31-fold slower than monomeric C6. Fitting the dose–response of C6_2_ with hHv1 at +40 mV to Eq. **1** gave a *K*_i_ of 631 ± 52 pM with a Hill coefficient of 1.01 ± 0.03 (Fig. 8*F* and Table 1). Supporting the specificity of C6_2_ for hHv1, CiHv1 was insensitive to 1 μM C6_2_ (*SI Appendix*, Fig. S12*A*), despite its homology to hHv1. Similarly, 1 μM C6_2_ did not inhibit the human voltage-gated potassium and sodium channels hKv1.3 and hNav1.5 (*SI Appendix*, Fig. S12 *B* and *C*), which are also naturally expressed in human neutrophils. The dose–response curve of C6_2_ at 0 mV showed a *K*_i_ of 41 ± 3 pM with a Hill coefficient of 1.07 ± 0.03 (*SI Appendix*, Fig. S9 and Table 1).

A homology model of C6_2_ and the down-state hHv1 was used to build a structural model of the C6_2_–hHv1 complex using MD simulations (*Materials and Methods*). The C6_2_–hHv1 complex in two orientations showing two C6 molecules (orange) are connected by the linker of 10 residues, and, therefore, both hHv1 subunits (cyan and blue) can bind a C6 epitope simultaneously (Fig. 8*G*).

## Discussion

### State-Dependent Binding of C6.

The role that hHv1 plays in acid extrusion and membrane-potential compensation place the channel in the midst of important human physiological processes and classify it as a promising pharmacological target (2). Here, we study inhibition of hHv1 by the specific designer toxin C6, which we created to delineate the roles of the hHv1 in sperm and neutrophils, in order to better understand the structural-mechanistic basis for C6 action and thereby its potential as a drug lead. Because C6 targets the hHv1 VSDs, binding is voltage-dependent, and inhibition is only partial at both 0 mV and +40 mV with affinities of *K*_i_ = 1.5 nM and 31 nM, respectively (Fig. 1*D* and Table 1). To characterize the molecular basis for voltage-dependent blockade, we used mutational scans to identify the residues on C6 and the channel that mediate binding, built a model for the C6–hHv1 complex in the closed state, validated the model by confirming predicted pairwise contacts on the interaction surface, and designed C6_2_ based on the model, a variant that fully suppresses the channel at depolarized potentials with picomolar affinity.

As observed for natural gating-modifier toxins (11, 12), we demonstrate that depolarization induces a change from the closed-channel conformation that binds C6 with high affinity to states that accelerate C6 dissociation, ∼15-fold faster at test pulses to +40 mV compared to 0 mV (Table 1). In keeping with this observation, a mutation in the S4 that favors the closed state (hHv1-R211S) slows C6 dissociation by ∼28-fold compared to WT channels, whereas a mutation that favors the open state (hHv1-R211S) accelerates C6 dissociation by ∼9-fold. Whereas C6 shows high affinity for the closed channel and positive cooperativity in binding, as the voltage becomes more depolarized, toxin association is slower, dissociation is faster, and negative cooperativity is observed, with a concomitant decrease in the extent of maximal blockade; thus, 250 nM C6 inhibits 90% of the current at −20 mV and 77% at 0 mV, due to a decrease in the on-rate of 1.2-fold and an increase in the off-rate of 2-fold, across this small change in potential (Fig. 1*C*). We produced a model of the C6–hHv1 complex by MD simulations based on studies of residue interaction identified in the channel S3–S4 loop (Fig. 2) and on C6 in its tethered form (Fig. 4), the latter a strategy that we have applied to identify important residues in other toxin-channel complexes (13). Toxin-depletion experiments and MD simulations (Fig. 5) revealed the importance of membrane lipids in C6 binding to hHv1. The model of the C6–hHv1 complex allows rationalization of state-dependent C6 affinity changes using MD simulations (*SI Appendix*, Fig. S10) and comparison to the mechanism of action of natural ICK toxins.

### Inhibition by C6 Compared to Natural ICK Toxins.

The mechanism of C6 blockade appears to have at least one similarity and two differences with natural ICK toxins that act on voltage-gated K^+^ and Na^+^ channels and TRPV1 (16, 32, 41). Like ICK spider toxins VSTx1 and HaTx (31, 37), C6 partitions into membranes by interaction with anionic lipids. Our MD simulations predict that basic residues in C6 form hydrogen bonds with lipid phosphate, ester oxygen atoms, and hydroxyl oxygen atoms in the head groups of anionic lipids (*SI Appendix*, Fig. S5*I*). The electrostatic interactions of C6 basic residues and the anionic lipids stabilize adhesion of C6 onto the surface of the membrane, enabling formation of extensive hydrogen-bond interactions of C6 residues and lipid polar head groups and insertion of bulky C6 hydrophobic side chains into the hydrophobic core of the membrane lipids (Fig. 5*D*). These interactions restrain the orientation of C6 in the membrane and its binding interface with the hHv1 channel.

The first apparent difference in the binding mechanism of C6 and natural ICK toxins is seen in the loop regions that the toxins use for membrane partitioning. Here, we compare C6 with findings for DkTx and ProTx2 visualized in complexes with TRPV1 and Nav1.7 channels, respectively, in a membrane-like environment using cryoelectron microscopy (16, 32). Whereas C6 uses two hydrophobic motifs locating in loop 4 (W25–F28) and the C terminus (V37–W38) for membrane partition (Fig. 5*D* and *SI Appendix*, Fig. S5*A*), DkTx activates TRPV1 by inserting both loop 4, where it carries F27, and loop 2, where it carries the bulky hydrophobic residue W11 (corresponding to C6-D13) (*SI Appendix*, Fig. S5*E*). When ProTx2 blocks Nav1.7, hydrophobic residues in loop 1, loop 4, and the C terminus anchor the toxin via four Trp residues (W5, W7, W24, and W30) in a double-stranded, antiparallel β-sheet that runs parallel to the membrane (*SI Appendix*, Fig. S5*F*) (32). These differences in membrane partitioning result in different lipid-insertion orientations and channel-binding orientations for the two native toxins and C6.

A second difference is that C6 binds to residues in the S3–S4 loop of hHv1 that project out toward the lipid membrane (Fig. 6), while the natural ICK toxins ProTx2 and HwTx-IV in complex with Nav1.7 insert positively charged residues into the extracellular crevice formed by the transmembrane segments of the channel VSD (32, 41). Thus, two basic residues in ProTx2 form salt bridges with negatively charged residues in the extracellular crevice of Nav1.7 VSD2 via the cleft between S2 and S3 and antagonize the movement of the gating-charge residues in the S4 to impede channel activation (*SI Appendix*, Fig. S11*A*) (32). Similarly, HwTx-IV inhibits Nav1.7 by inserting three basic residues into the extracellular crevice of VSD2 to form salt bridges with negatively charged residues in the S1–S3 to stabilize a closed-channel state (32, 41). A structural comparison shows that the S3 helix of hHv1 is one helical turn longer than the VSD2 in Nav1.7 (*SI Appendix*, Fig. S11*B*), blocking the cleft between S2 and S3 (*SI Appendix*, Fig. S11*C*), suggesting that toxins like ProTx2 are unable to reach the extracellular crevice of the hHv1 VSDs (32). In contrast, our C6–hHv1 model shows that hydrophobic interactions between C6-F28 and hHv1-L200 and between C6-W38 and hHv1-V187 are within the lipid membrane (Fig. 6*B*), while other C6 residues (K31, M33, and R36) interact with the extracellular S3–S4 loop of hHv1, so that C6 associates with the closed state of hHv1 on the lipid-facing surface of the channel formed by S3, S4, and the S3–S4 loop.

Three pairs of toxin-channel interactions suggested by the model were validated by mutant-cycle analysis (Fig. 7*I*), and these serve to rationalize the voltage-dependent blocking mechanism. In the model, the S4 helices of hHv1 are in the down position, placing V187 and L200 adjacent to W38 and F28 in C6, respectively. The distance between E192 and E196 in the S3–S4 loop (16 Å) matches the distance between K31 and R36 in C6 (15 Å), enabling a favorable electrostatic interaction (Fig. 6*B*). We hypothesize that C6 is destabilized on its closed-state binding site by depolarization because upward movement of S4 by one helical turn to the active conformation is predicted to cause a steric clash that breaks interactions like the salt bridge between C6-K31 and hHv1-E196 (*SI Appendix*, Fig. S10). Our functional data show that mutations of residues G199 and I202 change the binding affinity of C6 significantly in opposite directions: G199C enhances inhibition by C6, and I202C reduces inhibition (Fig. 2). In contrast to the five critical channel residues that we conclude make direct contact with C6 (Fig. 6*B*), G199 and I202 are away from the proposed hHv1–C6 binding interface in both our model and the AlphaFold model (*SI Appendix*, Fig. S6 *D* and *E*). In a recent study of the ICK spider toxin ProTx2 (32), Xu et al. propose that a phenylalanine in the S3 of domain II in the human sodium channel Nav1.7 underlies its sensitivity to the toxin compared to other Nav channel subtypes that bear glycine in the position, a residue that has lower helical propensity, thereby destabilizing the DII–S3 helix. The G199C mutation of hHv1 may play a similar role by increasing the helical propensity of the extracellular end of S4 to influence C6 binding to hHv1. In addition, G199 and I202 are in contact with L117 and I121 on S1 of the other channel subunit in our dimeric hHv1 homology model (*SI Appendix*, Fig. S6*E*), where mutations might perturb the stability of the dimer to impact cooperative binding of C6.

### Bivalent C6 Is an Advanced Blocker for Suppressing hHv1.

We designed C6 to block hHv1 because previously reported blockers, including Zn^2+^ ions (42), HaTx (a spider toxin that blocks numerous K^+^ and Ca^2+^ channels) (9), and guanidinium derivatives that block from the inside of the membrane (10), are promiscuous and of low affinity, limiting their potential use in delineating the roles of hHv1 in physiology or as therapeutics. The case for hHv1 as a drug target is strong. While hHv1 is required to sustain the ROS production by neutrophils that is essential for clearance of bacterial infections by innate immune cells, excessive ROS production induces tissue damage, thrombosis, and red blood cell dysfunction, contributing, for example, to the severity of COVID-19 (43). Further, in the central nervous system, ROS production by microglia was lower in an Hv1 knockout mouse than in WT, and this decreased the damage of ischemic stroke (19), and in a spinal cord injury mouse model, the knockout prevented secondary damage after spinal cord injury (44). In addition, Hv1 is up-regulated in some B-cell malignancies and breast cancers, and this is correlated with an increased metastatic potential (20, 45).

The ability of C6 to suppress sperm capacitation and sustained release of inflammatory mediators, including ROS, from neutrophils (6) is likely due to high-affinity toxin binding to closed channels, the state favored at the resting membrane potential of the cells (*K*_d_ of 0.75 nM at −49 mV). While C6 stabilization of the closed state, and partial open-channel blockade, might be sufficient for some therapeutic applications, we sought a more potent bivalent blocker that would bind to both hHv1 subunits simultaneously and used our C6–hHv1 model as a guide to design C6_2_ (Fig. 8). C6_2_ is notable, first, because it can fully inhibit hHv1 at depolarized voltages (Fig. 8*D*). Second, the affinity of C6_2_ is ∼50-fold better than C6 at +40 mV (Figs. 1*D* and 8*E*). Considering as an example the significant membrane depolarization of human neutrophils during the respiratory burst (17), both attributes of C6_2_ can be expected to enhance its efficacy as a suppressant of hHv1-mediated, neutrophil-associated inflammatory pathophysiology.

## Materials and Methods

An extended version of *Materials and Methods* is available in *SI Appendix*, *SI Materials and Methods*.

### Molecular Biology.

Human Hv1 (NM_001040107) tagged with a teal fluorescent protein was constructed by using gBlock gene fragments and inserted into the pMAX+ vector, as reported (6). Per our prior reports (13), T-C6 was constructed by replacing the sequence of lynx1, a natural tethered nicotinic acetylcholine receptor peptide modulator, by the sequence of C6 (with N terminus at front) in frame between the trypsin secretory signal sequence and a six-residue flexible linker containing a Gly–Asn repeat. The whole sequence of T-C6 was cloned into the pCS2+ plasmid vector that has an SP6 promoter for in vitro transcription of cRNA. T-C6_46_ has a 46-residue flexible linker, in which a c-Myc epitope tag is incorporated. T-C6_2_ was constructed in the backbone of T-C6 by replacing the C6 sequence with nucleotides encoding two C6 peptides linked by a flexible linker of either 5 or 20 Gly–Asn repeats using gBlocks. Point mutations of hHv1 and T-C6 were introduced by using the QuikChange Site-Directed Mutagenesis Kit. The sequences of all constructs were confirmed by DNA sequencing.

### Toxin Peptide Synthesis and Purification.

C6 peptide (GenBank accession no. AZI15804) and its variants, including C6-extN and C6-extC, were synthesized by CSBio. Peptides were dissolved in external solutions for electrophysical recordings before use.

### Whole-Cell Patch Clamp.

Proton currents passed by hHv1 were recorded in whole-cell mode by using an Axopatch 200B amplifier. Stimulation and data collection were done with a Digidata1322A and the pCLAMP 10 software (Molecular Devices). Cells were perfused with an external solution comprising 100 mM Hepes, 100 mM NaCl, and 10 mM glucose at pH 7.5. Pipettes with resistances between 3 and 5 MΩ were filled with 100 mM Bis-Tris buffer, 100 mM NaCl, and 10 mM glucose at pH 6.5. Capacitance was subtracted online. Sampling frequency was 10 kHz with filtering at 1 kHz. C6 block and unblock, current–voltage relationships, conductance–voltage relationships, and the dose–response curves were determined as described in *SI Appendix*, *SI Materials and Methods*.

### Two-Electrode Voltage Clamp.

*Xenopus laevis* stage VI oocytes were selected and injected with 10 ng of cRNA encoding hHv1 in the laboratory pMAX+ vector, as before (13, 46). To study the blocking effect of T-C6 (or variants), cRNAs for T-C6 (or variants) and hHv1 were mixed and coinjected into the oocytes. The recording solution was 60 mM NaCl, 1 mM MgCl_2_, 2 mM CaCl_2_, 120 mM Hepes, and 40 mM sucrose at pH 7.2. To prevent changes in intracellular pH due to the proton efflux, oocytes were injected with 50 nL of 1 M Hepes (pH 7.2) to produce ∼100 mM Hepes in the cytosol 30 min before recording. Currents were recorded 2 d after cRNA injection by using an Oocyte clamp amplifier (OC-725C, Warner Instruments) with electrodes filled with 3 M KCl and resistances of 0.3 to 1 MΩ.

### ELISA.

Surface expression of T toxins were quantitated by using ELISA as described (13). Oocytes injected with cRNAs of T-C6 variants bearing the c-Myc tag were blocked with bovine serum albumin and then bound with c-Myc–Tag monoclonal antibody conjugated with horseradish peroxidase (1 μg/mL) (Invitrogen). Oocytes were washed and then incubated with 50 μL of 1-Step Ultra TMB–ELISA solution (Thermo Fisher Scientific). The reaction was stopped by adding 50 μL of 2 M H_2_SO_4_. Surface ELISA signals were quantitated at 450 nm.

### Toxin-Depletion Assay.

POPC and POPG were dried from a chloroform solution under a nitrogen stream. The dried lipids were rehydrated in a buffer comprising 10 mM Hepes, 150 mM NaCl, 2 mM CaCl_2_, and 1 mM ethylenediaminetetraacetic acid (EDTA) at pH 7.5. The resulting dispersions were extruded through 100-nm pore-size polycarbonate filters (Millipore) and mixed at a POPC:POPG = 3:1 ratio to form LUVs. Varying concentrations of POPC:POPG LUVs or POPG-only LUVs were added to an aqueous C6 solution (final concentration of C6 was 50 μM in 200 μL) and incubated with gentle agitation for 30 min at room temperature. LUVs were separated by high-speed centrifugation (100,000 × *g*, 20 min). C6 toxin remaining in the aqueous phase was determined by using HPLC. Depletion experiments with native cell membranes are described in *SI Appendix*, *SI Materials and Methods*.

### Simulation Systems and MD Simulations.

The homology model of C6 was constructed with the MODELER program (47) using the NMR structure of the natural ICK spider toxin Mu-agatoxin-Aa1a as the template (PDB ID code 1EIT), as described in *SI Appendix*, *SI Materials and Methods*. The bilayers of POPC:POPG (3:1) or pure POPC lipids were built by using the web service CHARMM-GUI (48). The six cysteine residues of C6 were patched to form three disulfide bonds using the psfgen plugin of VMD (49). The C6 was placed on the extracellular side of the bilayer and then solvated with 100 mM KCl solution using VMD. The two final C6-membrane systems were minimized and equilibrated by using NAMD (50) and then simulated for 1 μs on the special-purpose computer Anton2 (51). The starting system of the C6–hHv1 complex was constructed by using the equilibrated C6 structure and the transmembrane region (residues G90 to I216) of the resting-state dimeric hHv1 model adopted from our previous study (23) using VMD. The hHv1 homology model was inserted into a bilayer of POPC:POPG (3:1) lipids, and two C6 molecules were placed on the extracellular side of hHv1 with the F28-containing loop (loop 4) facing the lipids and the potential binding interface facing the S3–S4 loop of each monomer of the channel. A stepwise target MD-simulations protocol (13) was used to refine the complex with distance restraints between C6–lipids and C6–hHv1 using NAMD, as described in *SI Appendix*, *SI Materials and Methods*. The “up” state of the hHv1 model was built by moving the S4 helix of the down state one helical turn outward, while leaving the S1–S3 helices unchanged, as in our previous study (23). The C6_2_ model was built by using MODELER, as described in *SI Appendix*, *SI Materials and Methods*. The C6_2_ model was inserted into the C6–hHv1 system to replace the two C6 monomers and then simulated, as described in *SI Appendix*, *SI Materials and Methods*.

## Supplementary Material

Supplementary File

## Data Availability

All data needed to evaluate the conclusions in the paper are present in the paper and/or *SI Appendix*.

## References

[1] T. E. DeCoursey, Voltage-gated proton channels: Molecular biology, physiology, and pathophysiology of the H(V) family. Physiol. Rev. 93, 599–652 (2013). 23589829 10.1152/physrev.00011.2012 PMC3677779

[2] T. Seredenina, N. Demaurex, K. H. Krause, Voltage-gated proton channels as novel drug targets: From NADPH oxidase regulation to sperm biology. Antioxid. Redox Signal. 23, 490–513 (2015). 24483328 10.1089/ars.2013.5806 PMC4543398

[3] I. S. Ramsey, M. M. Moran, J. A. Chong, D. E. Clapham, A voltage-gated proton-selective channel lacking the pore domain. Nature 440, 1213–1216 (2006). 16554753 10.1038/nature04700 PMC4084761

[4] M. Sasaki, M. Takagi, Y. Okamura, A voltage sensor-domain protein is a voltage-gated proton channel. Science 312, 589–592 (2006). 16556803 10.1126/science.1122352

[5] B. Musset , Aspartate 112 is the selectivity filter of the human voltage-gated proton channel. Nature 480, 273–277 (2011). 22020278 10.1038/nature10557 PMC3237871

[6] R. Zhao , Role of human Hv1 channels in sperm capacitation and white blood cell respiratory burst established by a designed peptide inhibitor. Proc. Natl. Acad. Sci. U.S.A. 115, E11847–E11856 (2018). 30478045 10.1073/pnas.1816189115 PMC6294887

[7] R. Zhao , Direct activation of the proton channel by albumin leads to human sperm capacitation and sustained release of inflammatory mediators by neutrophils. Nat. Commun. 12, 3855 (2021). 34158477 10.1038/s41467-021-24145-1 PMC8219737

[8] V. V. Cherny, T. E. DeCoursey, pH-dependent inhibition of voltage-gated H(+) currents in rat alveolar epithelial cells by Zn(2+) and other divalent cations. J. Gen. Physiol. 114, 819–838 (1999). 10578017 10.1085/jgp.114.6.819 PMC2230650

[9] A. A. Alabi, M. I. Bahamonde, H. J. Jung, J. I. Kim, K. J. Swartz, Portability of paddle motif function and pharmacology in voltage sensors. Nature 450, 370–375 (2007). 18004375 10.1038/nature06266 PMC2709416

[10] L. Hong, M. M. Pathak, I. H. Kim, D. Ta, F. Tombola, Voltage-sensing domain of voltage-gated proton channel Hv1 shares mechanism of block with pore domains. Neuron 77, 274–287 (2013). 23352164 10.1016/j.neuron.2012.11.013 PMC3559007

[11] L. R. Phillips , Voltage-sensor activation with a tarantula toxin as cargo. Nature 436, 857–860 (2005). 16094370 10.1038/nature03873

[12] J. Wang , Mapping the receptor site for alpha-scorpion toxins on a Na+ channel voltage sensor. Proc. Natl. Acad. Sci. U.S.A. 108, 15426–15431 (2011). 21876146 10.1073/pnas.1112320108 PMC3174582

[13] R. Zhao, H. Dai, N. Mendelman, J. H. Chill, S. A. N. Goldstein, Tethered peptide neurotoxins display two blocking mechanisms in the K^+^ channel pore as do their untethered analogs. Sci. Adv. 6, eaaz3439 (2020). 32181366 10.1126/sciadv.aaz3439 PMC7056315

[14] C. J. Bohlen , A bivalent tarantula toxin activates the capsaicin receptor, TRPV1, by targeting the outer pore domain. Cell 141, 834–845 (2010). 20510930 10.1016/j.cell.2010.03.052 PMC2905675

[15] I. R. Chassagnon , Potent neuroprotection after stroke afforded by a double-knot spider-venom peptide that inhibits acid-sensing ion channel 1a. Proc. Natl. Acad. Sci. U.S.A. 114, 3750–3755 (2017). 28320941 10.1073/pnas.1614728114 PMC5389327

[16] Y. Gao, E. Cao, D. Julius, Y. Cheng, TRPV1 structures in nanodiscs reveal mechanisms of ligand and lipid action. Nature 534, 347–351 (2016). 27281200 10.1038/nature17964 PMC4911334

[17] T. E. DeCoursey, During the respiratory burst, do phagocytes need proton channels or potassium channels, or both? Sci. STKE 2004, pe21 (2004). 15150421 10.1126/stke.2332004pe21

[18] M. A. Matthay , Acute respiratory distress syndrome. Nat. Rev. Dis. Primers 5, 18 (2019). 30872586 10.1038/s41572-019-0069-0 PMC6709677

[19] L. J. Wu , The voltage-gated proton channel Hv1 enhances brain damage from ischemic stroke. Nat. Neurosci. 15, 565–573 (2012). 22388960 10.1038/nn.3059 PMC3314139

[20] Y. Wang , Specific expression of the human voltage-gated proton channel Hv1 in highly metastatic breast cancer cells, promotes tumor progression and metastasis. Biochem. Biophys. Res. Commun. 412, 353–359 (2011). 21821008 10.1016/j.bbrc.2011.07.102

[21] J. Peng , The voltage-gated proton channel Hv1 promotes microglia-astrocyte communication and neuropathic pain after peripheral nerve injury. Mol. Brain 14, 99 (2021). 34183051 10.1186/s13041-021-00812-8 PMC8240390

[22] Q. Zhang , Inhibiting Hv1 channel in peripheral sensory neurons attenuates chronic inflammatory pain and opioid side effects. Cell Res. 32, 461–476 (2022). 35115667 10.1038/s41422-022-00616-y PMC9061814

[23] Q. Li , Resting state of the human proton channel dimer in a lipid bilayer. Proc. Natl. Acad. Sci. U.S.A. 112, E5926–E5935 (2015). 26443860 10.1073/pnas.1515043112 PMC4640771

[24] V. Yarov-Yarovoy , Structural basis for gating charge movement in the voltage sensor of a sodium channel. Proc. Natl. Acad. Sci. U.S.A. 109, E93–E102 (2012). 22160714 10.1073/pnas.1118434109 PMC3258622

[25] S. B. Long, E. B. Campbell, R. Mackinnon, Voltage sensor of Kv1.2: Structural basis of electromechanical coupling. Science 309, 903–908 (2005). 16002579 10.1126/science.1116270

[26] Q. Li , Structural mechanism of voltage-dependent gating in an isolated voltage-sensing domain. Nat. Struct. Mol. Biol. 21, 244–252 (2014). 24487958 10.1038/nsmb.2768 PMC4116111

[27] A. D. Geragotelis , Voltage-dependent structural models of the human Hv1 proton channel from long-timescale molecular dynamics simulations. Proc. Natl. Acad. Sci. U.S.A. 117, 13490–13498 (2020). 32461356 10.1073/pnas.1920943117 PMC7306757

[28] R. Ranganathan, J. H. Lewis, R. MacKinnon, Spatial localization of the K+ channel selectivity filter by mutant cycle-based structure analysis. Neuron 16, 131–139 (1996). 8562077 10.1016/s0896-6273(00)80030-6

[29] S. Lise, C. Archambeau, M. Pontil, D. T. Jones, Prediction of hot spot residues at protein-protein interfaces by combining machine learning and energy-based methods. BMC Bioinformatics 10, 365 (2009). 19878545 10.1186/1471-2105-10-365 PMC2777894

[30] R. Zhao, S. A. N. Goldstein, “Tethered peptide toxins for ion channels” in Methods in Enzymology D. L. Minor Jr., H. M. Colecraft, Eds. (Academic Press, New York, 2021), vol. 645, pp. 203–224. 10.1016/bs.mie.2021.03.002 34120714

[31] M. Milescu , Tarantula toxins interact with voltage sensors within lipid membranes. J. Gen. Physiol. 130, 497–511 (2007). 17938232 10.1085/jgp.200709869 PMC2151668

[32] H. Xu , Structural basis of Nav1.7 inhibition by a gating-modifier spider toxin. Cell 176, 702–715.e14 (2019). 30661758 10.1016/j.cell.2018.12.018

[33] S. Y. Lee, R. MacKinnon, A membrane-access mechanism of ion channel inhibition by voltage sensor toxins from spider venom. Nature 430, 232–235 (2004). 15241419 10.1038/nature02632

[34] A. Gross, R. MacKinnon, Agitoxin footprinting the shaker potassium channel pore. Neuron 16, 399–406 (1996). 8789954 10.1016/s0896-6273(00)80057-4

[35] D. O. Omecinsky, K. E. Holub, M. E. Adams, M. D. Reily, Three-dimensional structure analysis of mu-agatoxins: Further evidence for common motifs among neurotoxins with diverse ion channel specificities. Biochemistry 35, 2836–2844 (1996). 8608119 10.1021/bi952605r

[36] B. Dang , Inversion of the side-chain stereochemistry of individual Thr or Ile residues in a protein molecule: Impact on the folding, stability, and structure of the ShK toxin. Angew. Chem. Int. Ed. Engl. 56, 3324–3328 (2017). 28194851 10.1002/anie.201612398 PMC5507063

[37] H. J. Jung , Solution structure and lipid membrane partitioning of VSTx1, an inhibitor of the KvAP potassium channel. Biochemistry 44, 6015–6023 (2005). 15835890 10.1021/bi0477034

[38] P. Hidalgo, R. MacKinnon, Revealing the architecture of a K+ channel pore through mutant cycles with a peptide inhibitor. Science 268, 307–310 (1995). 7716527 10.1126/science.7716527

[39] C. Bae , Structural insights into the mechanism of activation of the TRPV1 channel by a membrane-bound tarantula toxin. eLife 5, e11273 (2016). 26880553 10.7554/eLife.11273 PMC4764579

[40] A. J. Agwa, L. V. Blomster, D. J. Craik, G. F. King, C. I. Schroeder, Efficient enzymatic ligation of inhibitor cystine knot spider venom peptides: Using sortase A to form double-knottins that probe voltage-gated sodium channel Na_V_1.7. Bioconjug. Chem. 29, 3309–3319 (2018). 30148615 10.1021/acs.bioconjchem.8b00505

[41] G. Wisedchaisri , Structural basis for high-affinity trapping of the Na_V_1.7 channel in its resting state by tarantula toxin. Mol. Cell 81, 38–48.e4 (2021). 33232657 10.1016/j.molcel.2020.10.039 PMC8043720

[42] B. Musset , Zinc inhibition of monomeric and dimeric proton channels suggests cooperative gating. J. Physiol. 588, 1435–1449 (2010). 20231140 10.1113/jphysiol.2010.188318 PMC2876801

[43] M. Laforge , Tissue damage from neutrophil-induced oxidative stress in COVID-19. Nat. Rev. Immunol. 20, 515–516 (2020). 32728221 10.1038/s41577-020-0407-1 PMC7388427

[44] M. Murugan , The voltage-gated proton channel Hv1 contributes to neuronal injury and motor deficits in a mouse model of spinal cord injury. Mol. Brain 13, 143 (2020). 33081841 10.1186/s13041-020-00682-6 PMC7574559

[45] M. Capasso , HVCN1 modulates BCR signal strength via regulation of BCR-dependent generation of reactive oxygen species. Nat. Immunol. 11, 265–272 (2010). 20139987 10.1038/ni.1843 PMC3030552

[46] R. Zhao , Designer and natural peptide toxin blockers of the KcsA potassium channel identified by phage display. Proc. Natl. Acad. Sci. U.S.A. 112, E7013–E7021 (2015). 26627718 10.1073/pnas.1514728112 PMC4687576

[47] A. Sali, T. L. Blundell, Comparative protein modelling by satisfaction of spatial restraints. J. Mol. Biol. 234, 779–815 (1993). 8254673 10.1006/jmbi.1993.1626

[48] S. Jo, T. Kim, V. G. Iyer, W. Im, CHARMM-GUI: A web-based graphical user interface for CHARMM. J. Comput. Chem. 29, 1859–1865 (2008). 18351591 10.1002/jcc.20945

[49] W. Humphrey, A. Dalke, K. Schulten, VMD: Visual molecular dynamics. J. Mol. Graph 14, 33–38 (1996). 8744570 10.1016/0263-7855(96)00018-5

[50] J. C. Phillips , Scalable molecular dynamics with NAMD. J. Comput. Chem. 26, 1781–1802 (2005). 16222654 10.1002/jcc.20289 PMC2486339

[51] D. E. Shaw , (2014) “Anton 2: Raising the bar for performance and programmability in a special-purpose molecular dynamics supercomputer” in *Proceedings of the International Conference for High Performance Computing, Networking, Storage and Analysis* (IEEE Press, Piscataway, NJ), pp. 41–53.

