# Supplementary material for: Molecular determinants of inhibition of the human proton channel hHv1 by the designer peptide C6 and a bivalent derivative
Source: Proc Natl Acad Sci U S A. 2022 Jun 1;119(23):e2120750119. doi: 10.1073/pnas.2120750119 (PMC9191634; doi:10.1073/pnas.2120750119)
Supplement: Supplementary File [file pnas.2120750119.sapp.pdf]

Supplemental information for:

Molecular determinants of inhibition of the human proton channel hHv1 by the designer peptide C6 and a bivalent derivative

Ruiming Zhao[1+], Rong Shen[2+], Hui Dai[1], Eduardo Perozo[2] and Steve A.N. Goldstein[1]

[1]Departments of Pediatrics, Physiology & Biophysics, and Pharmaceutical Sciences, Susan and Henry Samueli College of Health Sciences, University of California, Irvine, CA 92697, USA

[2]Department of Biochemistry and Molecular Biology, Gordon Center for Integrative Science, University of Chicago, Chicago, IL, 60637, USA

Contents

Table S1. Effect of hHv1 point mutations on block by C6

Table S2. Effects of critical mutations in T-C6 and T-$C6_{46}$ on block of hHv1

Table S3. K_i for combinations of toxin-channel pairs shown in Fig. 7A-F

Fig. S1. The primary sequence and intramolecular disulfide bonds of C6

Fig. S2. Construction of T-C6 and T-$C6_{46}$

Fig. S3. T-$C6_{46}$ screening mutagenesis and surface expression determination

Fig. S4. Lipid membrane partitioning of C6 peptide

Fig. S5. Homology model of C6 and its interaction with lipids

Fig. S6. Experimental structures and models of Hv1 proton channels

Fig. S7. Salt bridge interactions between C6 and hHv1

Fig. S8. Purification of bivalent C6 by HPLC

Fig. S9. Dose–response relationship for $C6_2$ inhibition of hHv1 at 0 mV

Fig. S10. C6 preferably interacts with the closed "down" state of hHv1

Fig. S11. C6 and ProTx2 interact differently with their target channels

Fig. S12. $C6_2$ does not inhibit CiHv1, hKv1.3 or hNav1.5 channels

Supplemental Information Materials and Methods

Supplemental Information References

Note: Supplemental PDB format coordinates for the models of C6, hHv1-C6 and hHv1-$C6_2$ in Figs. 5, 6 and 8 are in ModelArchive (modelarchive.org) with the accession codes: ma-1o3ga (C6), ma-rlflj (hHv1-C6) and ma-7mmcm (hHv1-$C6_2$).

Table S1. Effect of hHv1 point mutations on block by C6

WT hHv1 and hHv1 mutant channels were expressed in HEK293T cells and the conductance–voltage relationships (G–V) studied with pH_i = 6.5, pH_o = 7.5 and the inhibition constant (K_i) of C6 at +40 mV were determined by whole-cell patch clamp as in Fig. 1. Among the 28 channel mutants, we performed dose-response studies for 14 mutants (2 are shown in Fig. 1, 8 are shown in Fig. 2, and 4 are shown in Fig. 7) and determined the Hill coefficient by fitting to Eq 1, observing a range of 0.4 to 0.56, as reported below. Based on those studies, the K_i and the ratio of $K_{i\ mutation}$/ $K_{i\ WT}$, for the other 14 mutants were estimated using Eqs. 1 and 5 assuming the Hill coefficient to be the same as WT (0.48). The $V_{1/2}$ of G–V were determined by fitting to a Boltzmann function. Changes in the free energy of C6 blockade (ΔΔG) were calculated with Eqs. 5 and 6. Values are mean ± SEM; ND, not determined; n = 3-6 cells for each construct.

Channels	$V_{1/2}$ (mV)	K_i (nM)	K_i ratio (mut/WT)	ΔΔG (kcal/mol)	Hill coefficient
WT hHv1	15 ± 2	31 ± 3	1	0	0.48 ± 0.04
hHv1-I183C	14 ± 2	34 ± 3	1	0.05	ND
hHv1-L184C	5 ± 1	682 ± 62	22	1.7	ND
hHv1-D185C	49 ± 3	7 ± 1	0.23	-0.7	0.43 ± 0.07
hHv1-I186C	13 ± 2	43 ± 4	1	0.2	ND
hHv1-V187A	12 ± 2	3346 ± 417	108	2.6	0.49 ± 0.05
hHv1-L188C	13 ± 2	335 ± 26	11	1.3	ND
hHv1-L189A	15 ± 2	26 ± 2	0.84	-0.1	ND
hHv1-F190C	12 ± 2	19 ± 2	0.6	-0.3	ND
hHv1-Q191C	17 ± 2	338 ± 37	11	1.1	ND
hHv1-E192C	32 ± 3	14089 ± 1084	454	3.2	0.47 ± 0.03
hHv1-E192A	35 ± 3	17763 ± 1480	573	3.3	0.47 ± 0.03
hHv1-H193C	7 ± 2	1627 ± 146	53	2.1	0.5 ± 0.1
hHv1-Q194C	18 ± 2	183 ± 17	6	0.9	0.56 ± 0.04
hHv1-Q194A	20 ± 2	244 ± 21	8	1.1	0.56 ± 0.04
hHv1-F195C	11 ± 2	109 ± 10	4	0.7	ND
hHv1-E196C	21 ± 2	1823 ± 166	59	2.2	0.43 ± 0.03
hHv1-E196A	22 ± 2	1537 ± 139	50	2.1	0.43 ± 0.03

hHv1-A197C	16 ± 2	232 ± 21	7	1.1	ND
hHv1-L198C	11 ± 2	44 ± 4	1	0.2	ND
hHv1-G199C	16 ± 3	1.9 ± 0.2	0.06	-1.5	0.5 ± 0.1
hHv1-L200C	3 ± 1	2325 ± 211	75	2.3	0.49 ± 0.06
hHv1-L200A	5 ± 1	1571 ± 141	51	2.1	0.49 ± 0.06
hHv1-L201C	19 ± 2	109 ± 11	4	0.7	ND
hHv1-I202C	17 ± 2	899 ± 92	29	1.8	ND
hHv1-L203A	12 ± 2	8 ± 1	0.25	-0.7	ND
hHv1-L204C	25 ± 3	67 ± 5	2	0.4	ND
hHv1-R205N	-42 ± 3	161 ± 19	5	0.9	0.5 ± 0.1
hHv1-R211S	48 ± 3	0.6 ± 0.1	0.02	-2.1	0.4 ± 0.1

Table S2. Effects of critical mutations in T-C6 and T-C6$_{46}$ on block of hHv1

T-C6, T-C6$_{46}$ and variants were constructed as described in Materials and Methods and Fig. S2. hHv1 was expressed in *Xenopus* oocytes and inhibition by T-C6, T-C6$_{46}$ and variants were studied by two-electrode voltage clamp (TEVC) after injection of 10 ng of channel cRNA and 20 ng of tethered toxin cRNA. The $K_{i\ variant}/K_{i\ WT}$ ratios were estimated using Eq. 5 and assuming the same Hill coefficient as the WT. Values are means ± SEM; n = 12 oocytes for each construct.

**Tethered toxin variants**	**$K_{i\ variant}/K_{i\ WT}$ ratio**	
	**T-C6**	**T-C6$_{46}$**
**WT**	1	1
**S14A**	54	46
**K24A**	61	52
**F28A**	123	102
**K31A**	86	72
**M33A**	286	229
**R36A**	495	387
**W38A**	59	50

Table S3. ***K*i for combinations of toxin-channel pairs shown in Fig. 7A-F**

WT hHv1 and mutant hHv1 channels were expressed in HEK293T cells and the K_i and Hill coefficient (h) of C6 peptide and its variants were determined at +40 mV by whole-cell patch clamp as described in Fig. 1. Values are mean ± SEM; n = 3-6 cells for each condition.

K_i (µM)	WT hHv1	hHv1-V187A	hHv1-E192A	hHv1-Q194A	hHv1-E196A	hHv1-L200A
**C6**	0.031 ± 0.003 (h = 0.48 ± 0.04)	3.3 ± 0.4 (h = 0.49 ± 0.05)	18 ± 1 (h = 0.47 ± 0.03)	0.24 ± 0.02 (h = 0.56 ± 0.04)	1.5 ± 0.1 (h = 0.43 ± 0.03)	1.6 ± 0.1 (h = 0.49 ± 0.06)
**C6-F28A**	15 ± 1 (h = 0.42 ± 0.03)	357 ± 40 (h = 0.47 ± 0.03)	8086 ± 674 (h = 0.46 ± 0.03)	70 ± 9 (h = 0.53 ± 0.04)	371 ± 34 (h = 0.42 ± 0.03)	46 ± 7 (h = 0.47 ± 0.04)
**C6-K31A**	2.8 ± 0.2 (h = 0.42 ± 0.03)	273 ± 31 (h = 0.44 ± 0.03)	1640 ± 109 (h = 0.44 ± 0.03)	10 ± 1 (h = 0.51 ± 0.03)	6.2 ± 0.5 (h = 0.42 ± 0.03)	141 ± 18 (h = 0.44 ± 0.03)
**C6-R36A**	15 ± 1 (h = 0.51 ± 0.04)	1825 ± 166 (h = 0.48 ± 0.04)	52 ± 4 (h = 0.42 ± 0.03)	151 ± 22 (h = 0.48 ± 0.03)	1230 ± 95 (h = 0.42 ± 0.03)	3234 ± 359 (h = 0.42 ± 0.03)

Fig. S1. The primary sequence and intramolecular disulfide bonds of C6

C6 was isolated from a phage-display library by sorting on purified hHv1 protein (1). C6 has 41 residues that come from three natural parental spider toxins, ω-ACTX-Hv1b (residues S1 to C11 of C6), JZTX21 (residues A12 to C23 of C6), and Hm2 (residues K24 to D41 of C6). C6 has six cysteines that form three intramolecular disulfide bonds (C4-C18, C11-C23, and C17-C34) to create and stabilize an inhibitor cysteine knot (ICK) scaffold.

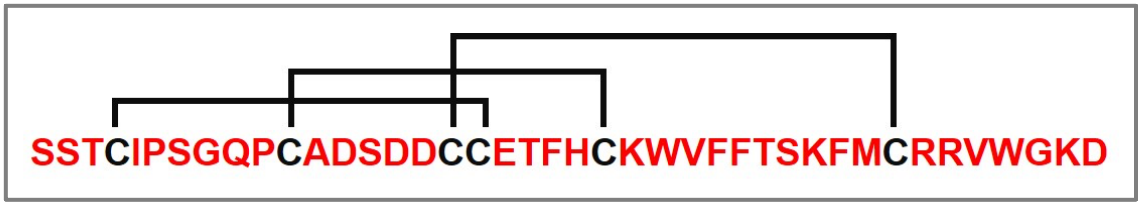

Fig. S2. Construction of T-C6 and T-C6$_{46}$

(A) Tethered C6 (T-C6) was constructed as chimeric fusion protein with a N-terminal secretory signal sequence (SP, gray), the C6 toxin sequence (red), a 6-residue hydrophilic flexible linker (3 Gly-Asn repeats), and a C-terminal GPI membrane anchor targeting sequence (orange). The T-C6 sequence was cloned into a pCS2+ plasmid vector using the restriction sites EcoR1 and Xho1 for synthesis of cRNA and expression in *Xenopus laevis* oocytes (Materials and Methods).

(B) T-C6$_{46}$ was constructed similarly to T-C6 except the 6-residue hydrophilic flexible linker was replaced by a 46-residue linker containing a c-Myc epitope tag (yellow) that allows quantification of surface expression of T-toxins using ELISA (2).

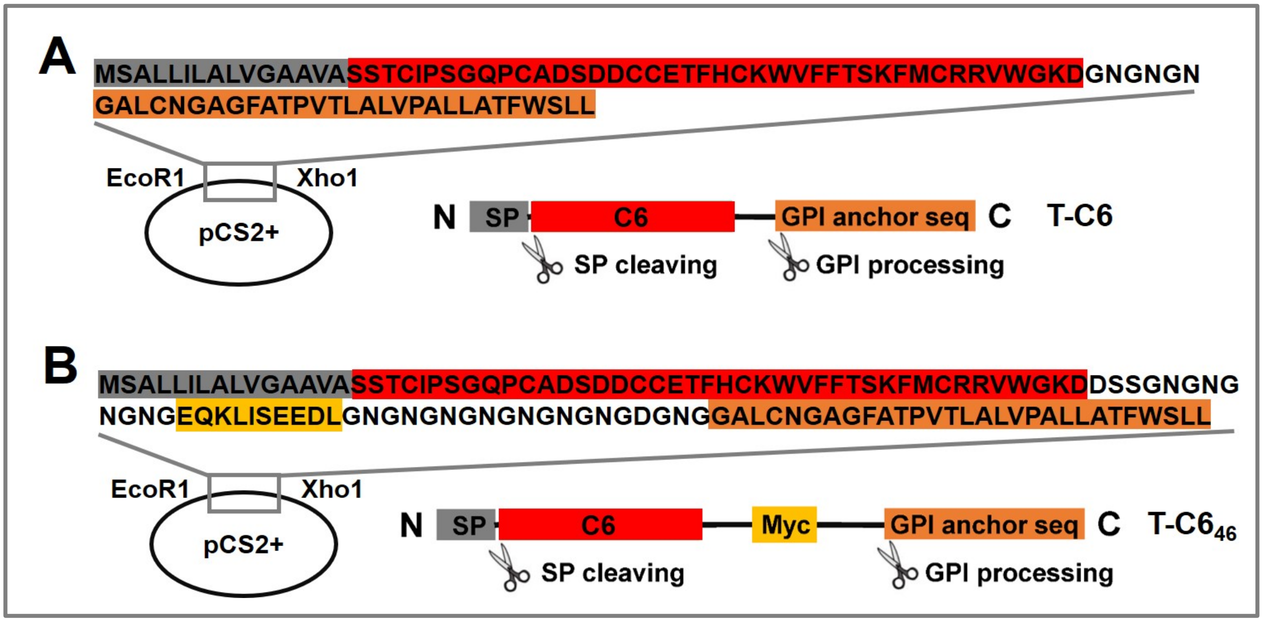

Fig. S3. T-C6$_{46}$ screening mutagenesis and surface expression determination

hHv1 was expressed in oocytes and the inhibition and surface expression of T-C6$_{46}$ and variants were studied by TEVC and ELISA, respectively, after injection of 10 ng of channel cRNA and 20 ng of tethered toxin cRNA (Materials and Methods). Values are means ± SEM; n = 12 oocytes for each condition.

Top: hHv1 currents at +80 mV measured as in Fig. 3 with T-C6$_{46}$ variants normalized to the unblocked condition (CTL). The affinity changes are reported in Table S2.

Bottom: ELISA performed as described in Materials and Methods with T-C6$_{46}$ variants. Control was without T-C6$_{46}$ cRNA injection.

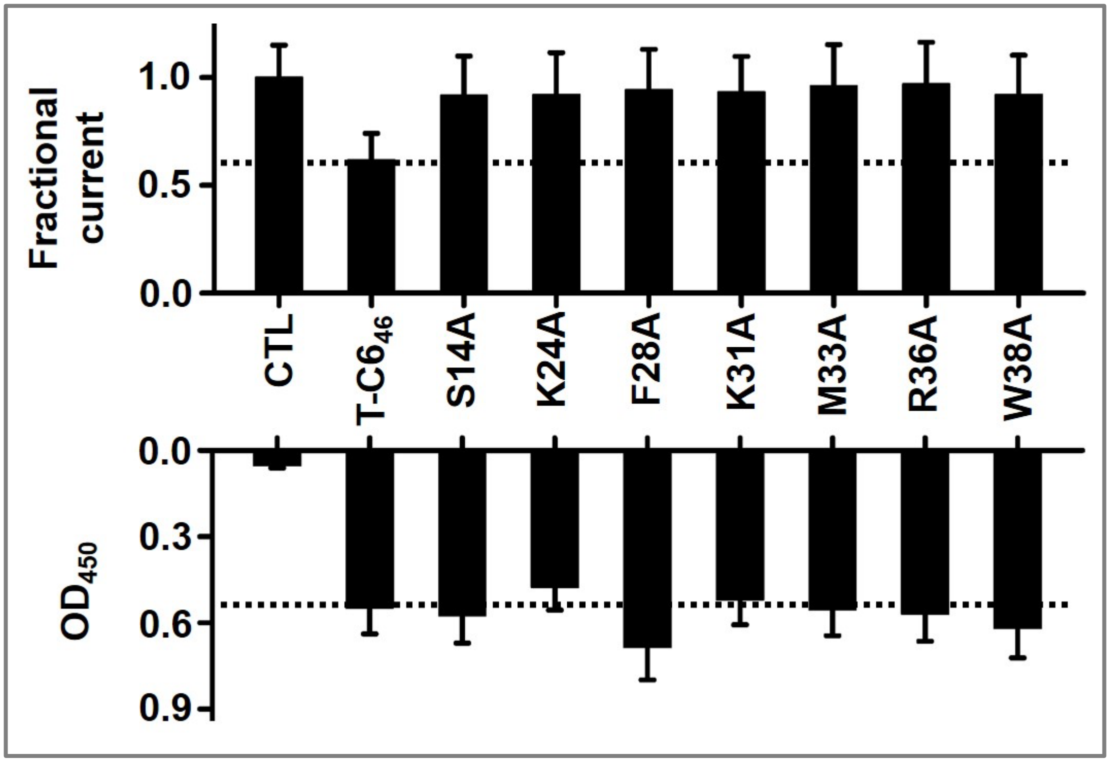

Fig. S4. Lipid membrane partitioning of C6 peptide

C6 peptide in solution was incubated with large unilamellar vesicles (LUVs), *Xenopus* oocytes, or HEK293T cells. C6 remaining in the aqueous phase was quantitated using HPLC as described in Materials and Methods.

(A) HPLC chromatogram shows free C6 that remains in the supernatant without (left) or with (right) incubation and ultracentrifugation with 0.5 mM POPC:POPG (3:1) LUVs.

(B) C6 partitioning into the plasma membranes of *Xenopus* oocytes (middle) and HEK293T (right). Defolliculated *Xenopus* oocytes (170) or 8.5×10^{6} HEK293T cells were judged to have similar levels of outer leaflet lipids and incubated with 0.5 mL C6 (12 µM) solution for 30 min before HPLC. C6 was depleted from the aqueous solution by oocytes and HEK293T whereas others have shown $AgTx_2$ was not depleted by oocytes (3).

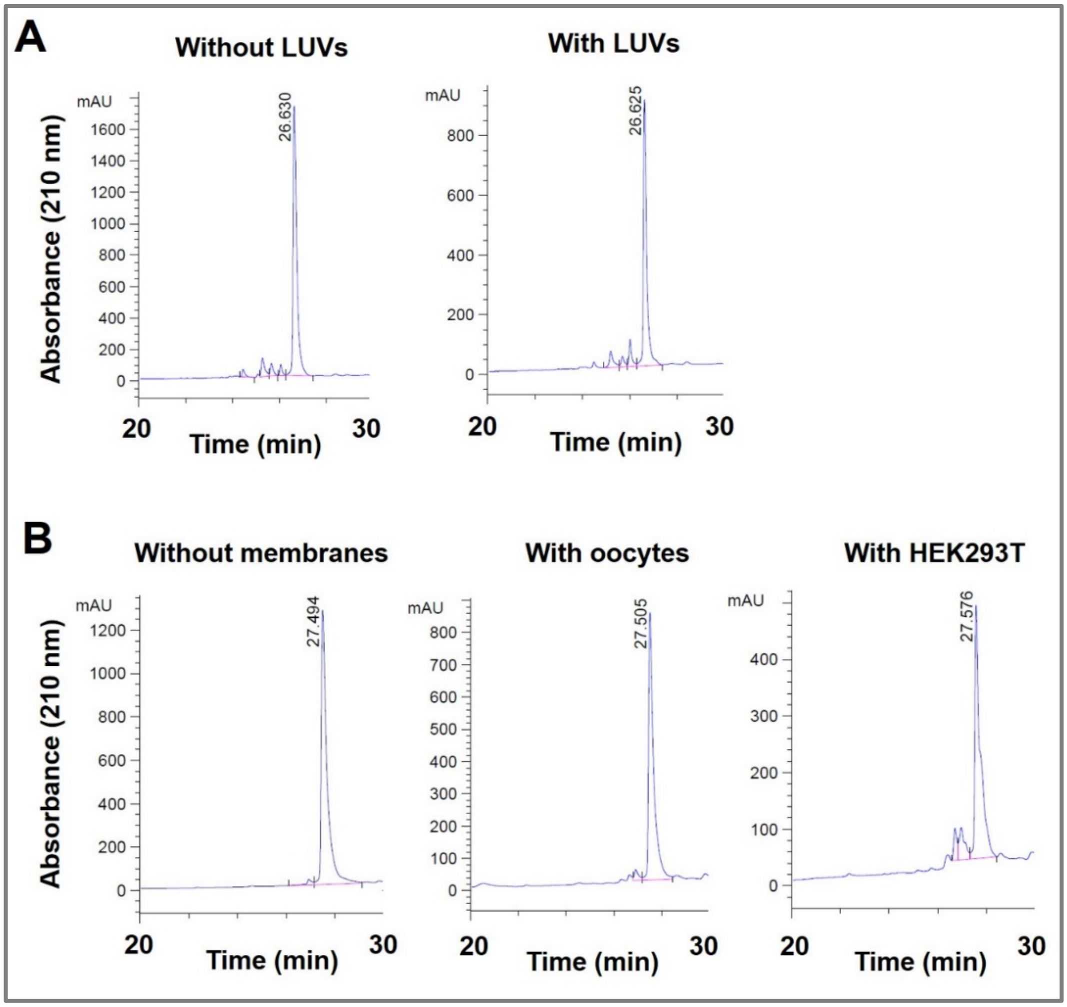

Fig. S5. Homology model of C6 and its interaction with lipids

ICK toxin structures were compared using the VMD program and the MD simulations of toxin-lipid interactions were performed using the NAMD program, as described in Materials and Methods.

(A) Sequence alignment of C6, Mu-agatoxin-Aa1a (template for C6 modeling), DkTx (first ICK motif), ProTx2 and HwTx-IV. The important toxin residues for membrane partitioning are labeled orange. N-terminus (NT), four loop regions and C-terminus (CT) of the ICK toxins are highlighted using underscores.

(B) Superimposition of NMR structures of Mu-agatoxin-Aa1a (PDB: 1EIT). The disulfide bonds and carbon alpha atoms of the cysteines are highlighted in stick and sphere representations, respectively. The structure used as the template in homology modeling is shown in solid representation.

(C) Backbone RMSF per residue of NMR structures of Mu-agatoxin-Aa1a (PDB: 1EIT). Data for the cysteine residues are highlighted in red.

(D) Superimposition of the template structure, Mu-agatoxin-Aa1a (yellow), and the homology model of C6 (cyan). The canonical disulfide bonds are highlighted in stick representation. C6 has three disulfide bonds (C4-C18, C11-C23, and C17-C34) and Mu-agatoxin-Aa1a has four.

(E) Superimposition of structures of DkTx (first ICK motif; PDB: 5IRX; magenta) and the homology model of C6 (cyan). The disulfide bonds and sidechain atoms of key residues influencing the penetration of toxins into lipids are highlighted in stick representation.

(F) Superimposition of structures of ProTx2 (PDB: 6N4I; red) and the homology model of C6 (cyan). The disulfide bonds and sidechain atoms of key residues influencing the penetration of toxin into lipids are highlighted in stick representation.

(G) Snapshot of C6 interacting with a POPC:POPG (3:1) lipid bilayer in the MD simulation. Phosphate atoms of lipids are shown in sphere representation (only part of the lipids are shown for clarity). The positively charged residues (K24, K34, R35, R36 and K40) and

negatively charged residues (D13, D15, D16, E19 and D41) of C6 are shown in stick and surface representation.

(H) Time series of the *z* coordinate of the center-of-mass of Cα atoms of the six cysteines of C6 (orange line), phosphate atoms in the upper and inner lipid leaflets (gray lines), and the heavy sidechain atoms of F28 (red line) and W38 (blue line) of C6 in the MD simulation system with a pure POPC lipid bilayer.

(I) Time series of the total number of hydrogen bonds formed by the five basic residues of C6 with water (cyan), phosphate oxygen and ester oxygen atoms of lipids (magenta), head group hydroxyl oxygen atoms (blue) of lipids, respectively, in the MD simulation system with a POPC:POPG (3:1) or pure POPC lipid bilayer.

(J) C6 partitioning into POPC LUVs was determined using a depletion assay as described in Fig. 5A and Fig. S4. The ratio of bound/free C6 (red) is plotted as a function of POPC LUVs concentration. n =3 for each condition. Error bars are smaller than symbols.

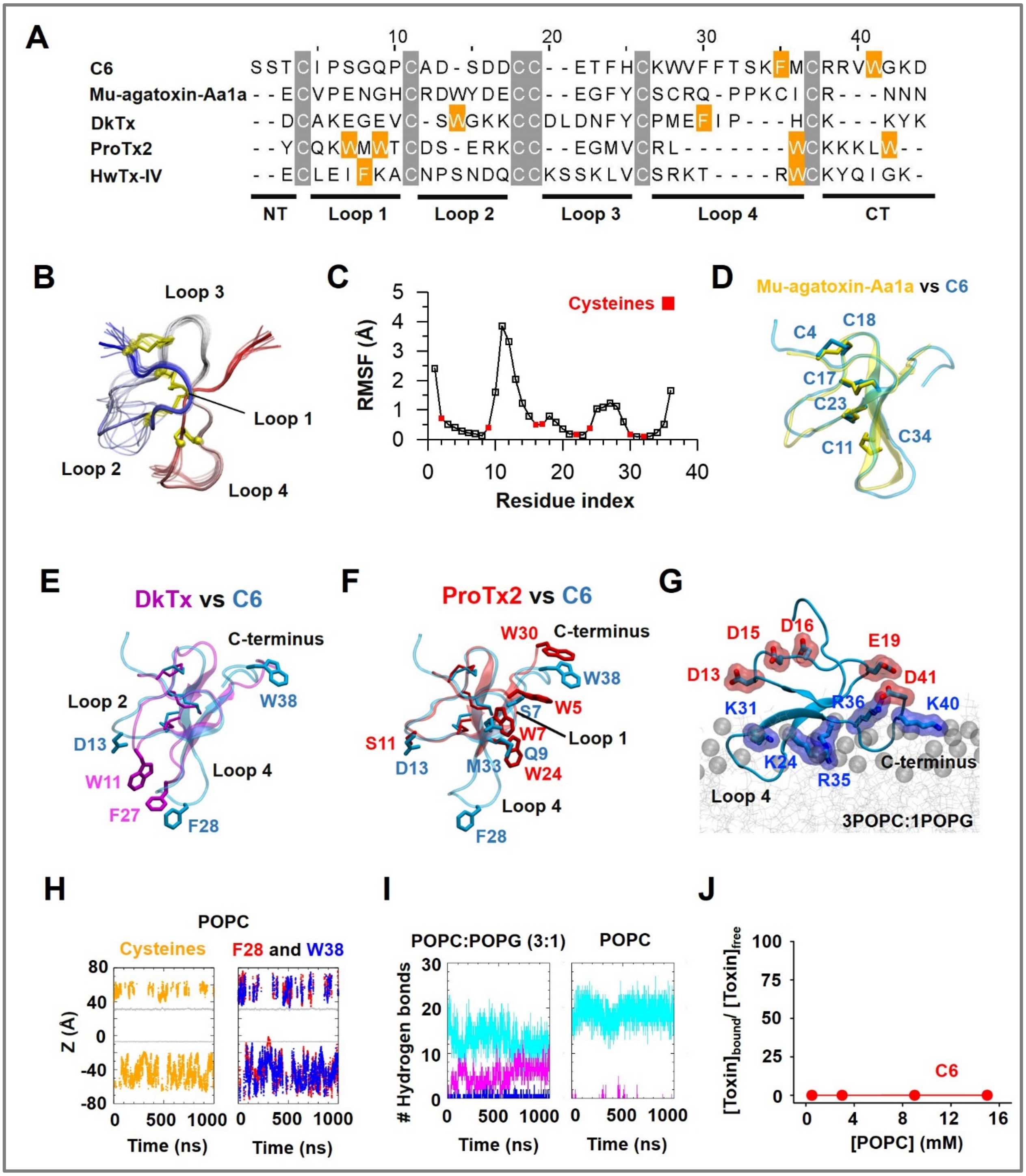

A
C6
Mu-agatoxin-Aa1a
DkTx
ProTx2
HwTx-IV
NT
Loop 1
Loop 2
Loop 3
Loop 4
CT
B
Loop 3
Loop 1
Loop 2
Loop 4
C
Cysteines
RMSF (Å)
Residue index
D
Mu-agatoxin-Aa1a vs C6
E
DkTx vs C6
C-terminus
Loop 2
W38
D13
W11
F27
F28
Loop 4
F
ProTx2 vs C6
W30
C-terminus
W38
W5
S7
W7
Loop 1
S11
D13
M33
Q9
W24
Loop 4
F28
G
D15
D16
E19
D13
D41
K31
R36
K40
K24
R35
C-terminus
Loop 4
3POPC:1POPG
H
POPC
Cysteines
F28 and W38
Z (Å)
Time (ns)
I
POPC:POPG (3:1)
POPC
Hydrogen bonds
Time (ns)
J
[Toxin]bound/ [Toxin]free
C6
[POPC] (mM)

Fig. S6. Experimental structures and models of Hv1 proton channels

(A) Superimposition of NMR structures of monomeric hHv1 (PDB: 5oqk). The NMR structure of model 1 is highlighted in ribbon representation.

(B) Crystallographic structure of chimeric mouse proton channel mHv1cc (PDB: 3wkv) determined in a homotrimer state; only the transmembrane region of one subunit was shown.

(C) Superimposition of the AlphaFold model of monomeric hHv1 (transparent) and one subunit of our dimeric homology model (solid) of hHv1.

(D) Close-up view of key residues for C6 binding in the AlphaFold model.

(E) Close-up view of key residues for C6 binding in our homology model. Residues L117 and I121 on S1 in the other subunit are shown in stick and surface representations.

(F) Side- and top-view of the “down” state Ci-VSD dimer (PDB: 4g80).

(G) Side- and top-view of our “down” state dimeric hHv1 model (Fig. 6A). Helices S1 and S4 form the dimer interface at the extracellular and intracellular sides, respectively.

(H) Side-view of our “down” state dimeric hHv1 model with the cytoplasmic coiled-coil region.

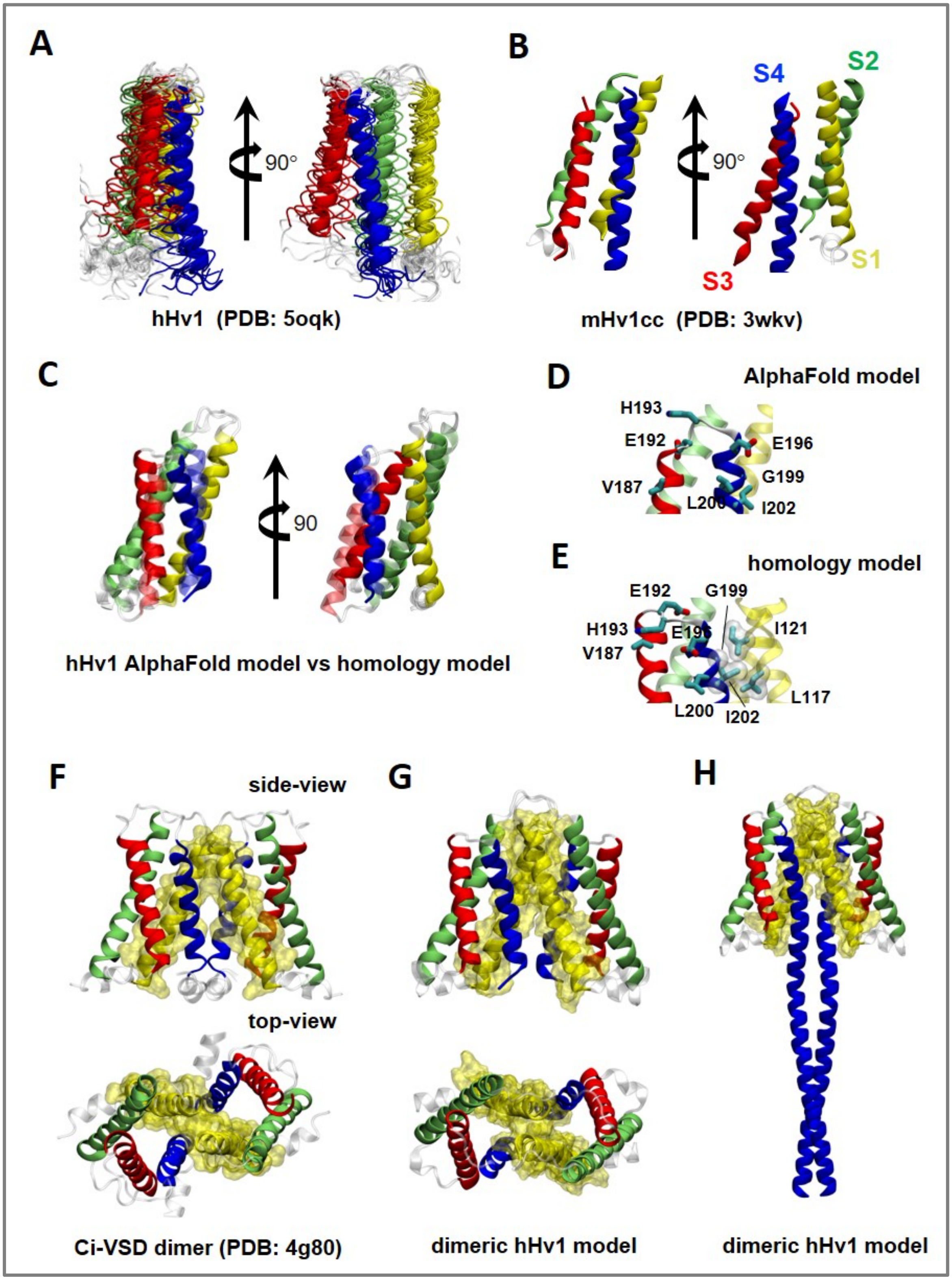
A
90°
hHv1 (PDB: 5oqk)
B
S4
S2
90°
S3
S1
mHv1cc (PDB: 3wkv)
C
90
hHv1 AlphaFold model vs homology model
D
AlphaFold model
H193
E192
E196
G199
V187
L200
I202
E
homology model
E192
G199
H193
I121
E196
V187
L117
L200
I202
F
side-view
G
H
top-view
Ci-VSD dimer (PDB: 4g80)
dimeric hHv1 model
dimeric hHv1 model

Fig. S7. Salt bridge interactions between C6 and hHv1

hHv1 was expressed in oocytes and inhibition by T-C6 (WT) and its variants were studied by TEVC after injection of 20 ng of the mixed cRNAs. hHv1 currents were measured at +80 mV as in Fig. 3 and normalized to the unblocked condition. Values are means ± SEM; n = 12 cells for each variant.

(A) Charged residue "swap" experiments demonstrate that C6-R36 directly interacts with hHv1-E192, and C6-K31 directly interacts with hHv1-E196.

(B) Snapshot of the C6-hHv1 complex showing two salt bridge interactions between C6 and hHv1. C6-K24 points away from hHv1 and interacts with the phosphate group of a bilayer lipid.

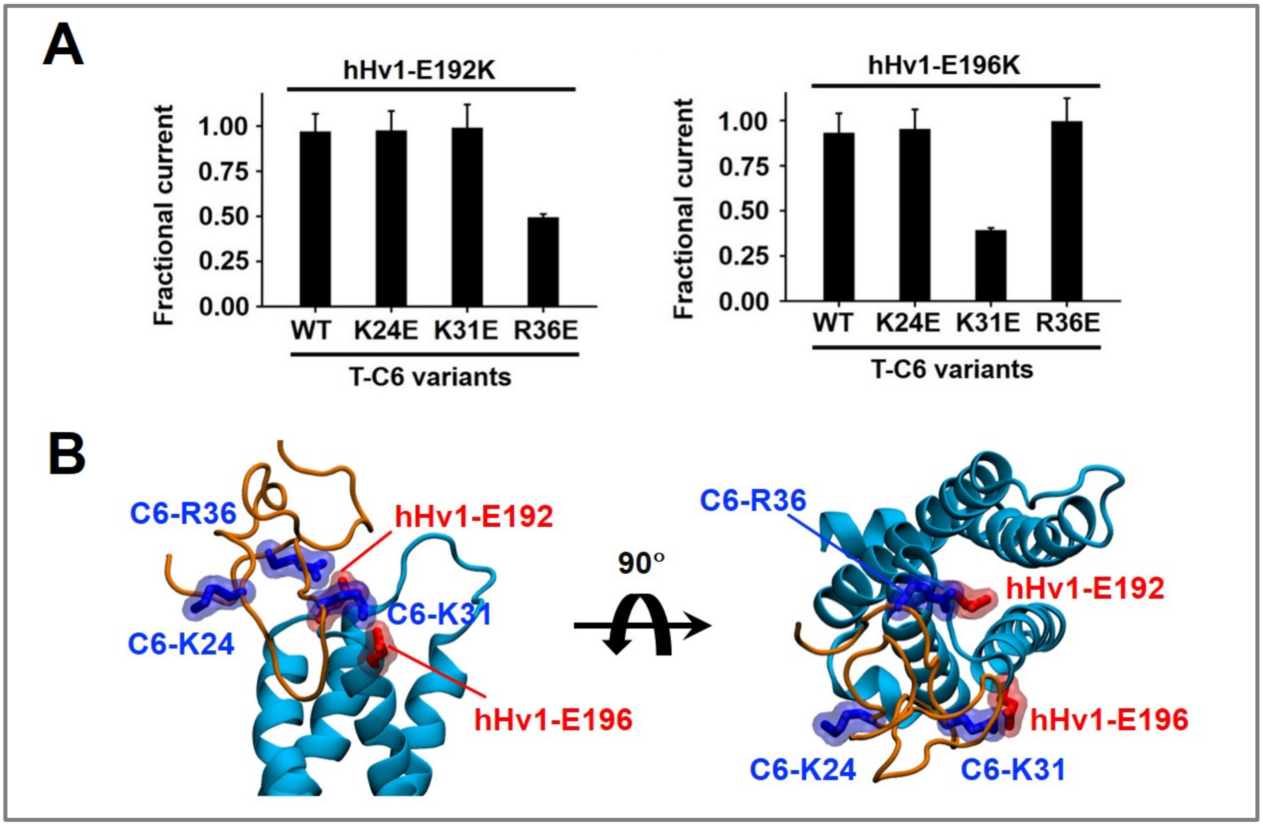

Fig. S8. Purification of bivalent C6 by HPLC

$C6_2$ peptide was made by ligating two C6 peptides (C6-extN and C6-extC) together using Sortase A as described in Fig. 8 and the Materials and Methods. Successfully ligated $C6_2$ was purified by HPLC.

(A) HPLC chromatogram of ligation mixture showing the reaction progress after 30 min. All starting materials (Sortase A, C6-extN and C6-extC) and the products of reaction (hydrolyzed C6-extC and bivalent C6) were observed. Hydrolyzed C6-extC is C6-extC with two C-terminal Gly residues cleaved by Sortase A.

(B) HPLC chromatogram of pure bivalent C6 that isolated from ligation reaction.

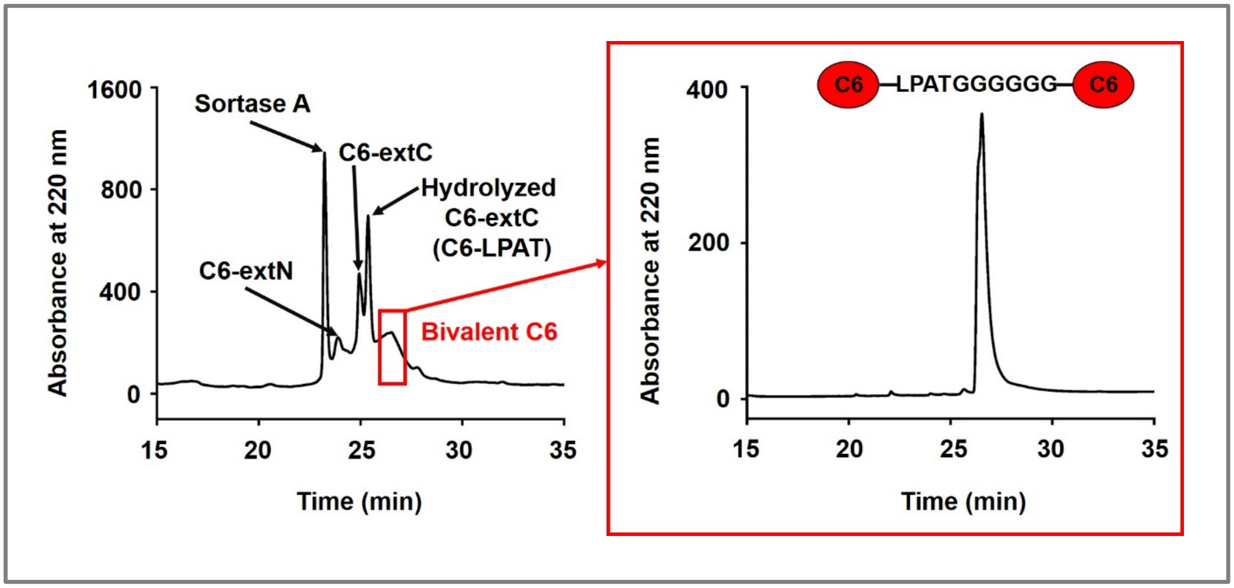

Fig. S9. Dose–response relationship for $C6_2$ inhibition of hHv1 at 0 mV

hHv1 was expressed in HEK293T cells and studied by whole-cell patch clamp using a holding voltage of −60 mV, 1.5-s test pulses, and a 10-s interpulse intervals, with pH_i = 6.5 and pH_o = 7.5. Values are mean ± SEM; n = 6 cells for each condition.

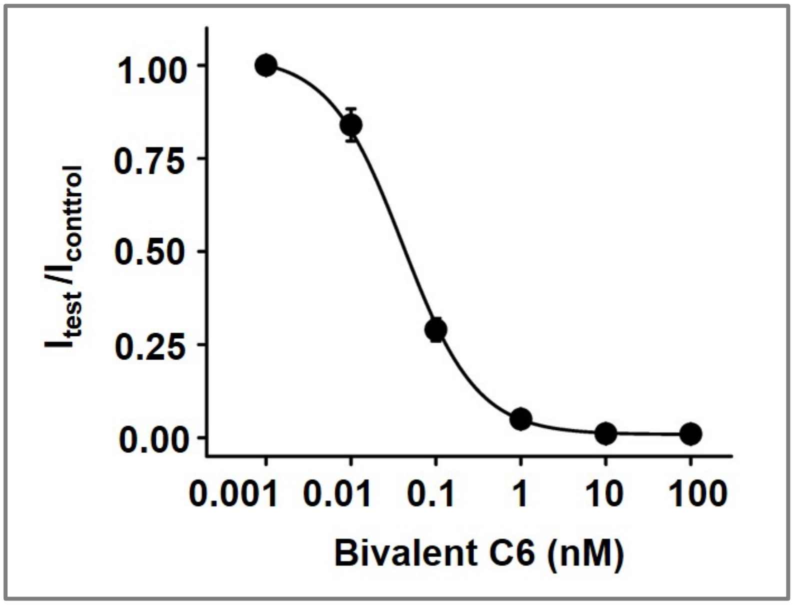

Fig. S10. C6 preferably interacts with the closed "down" state of hHv1

The "up" state model of hHv1 was generated as described in Materials and Methods and compared to the "down" state of hHv1 using the VMD program.

(A) C6 and the "down" state hHv1 complex model showing three critical residue-residue interactions (C6-F28 and hHv1-L200, C6-K31 and hHv1-E196, and C6-R36 and hHv1-E192) as confirmed by mutant cycle analysis (**Fig. 7**).

(B) A one helical turn upward movement of the S4 helix causes steric clashes between hHv1 and C6 as highlighted by the dashed oval (red), and breaks key interactions, notably, the salt-bridge between C6-K31 and hHv1-E196. For clarity, the "down" state hHv1 model is shown in transparent representation, and only the S4 helix and the S3-S4 loop of the activated "up" state hHv1 channel are shown.

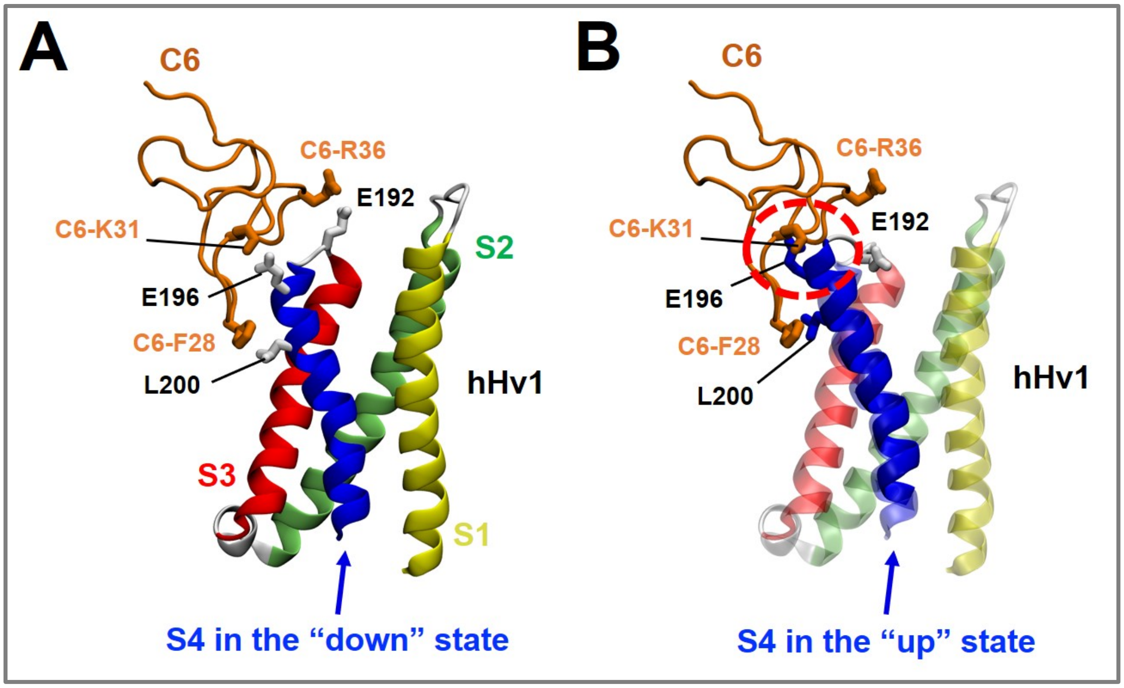

Fig. S11. C6 and ProTx2 interact differently with their target channels

The comparison of hHv1 homology model and the crystal structure of voltage-sensor domain 2 (VSD2) of Nav1.7-NavAb chimeric channel (PDB: 6N4I) was performed using the VMD program.

(A) ProTx2 binds directly on top of the S3 of Nav1.7-VSD2-NavAb to interact with the channel S2 span and S3-S4 loop. ProTx2 acts as an electrostatic gating modifier by stretching two basic residues (R22 and K26) into the extracellular crevice of VSD2 and forming salt bridges with negatively charged residues on S3 and S3-S4 loop (4).

(B) Superimposition of one subunit of hHv1 (solid) and Nav1.7-VSD2-NavAb (transparent), showing that the S3 helix of hHv1 is one helical turn longer than that of Nav1.7-VSD2-NavAb.

(C) Side views of ProTx2 superimposed on hHv1 based on the alignment between hHv1 and Nav1.7-VSD2-NavAb. The extension of S3 in hHv1 is a steric impediment to the binding of ProTx2 and prohibits its ready interaction with negatively charged residues inside the extracellular crevice of hHv1.

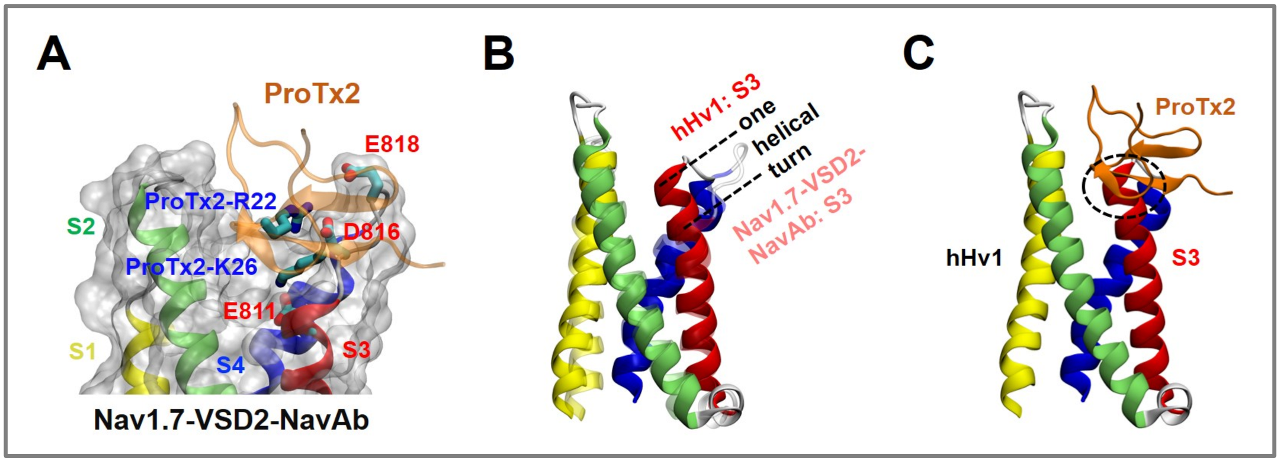

Fig. S12. $C6_2$ does not inhibit CiHv1, hKv1.3 or hNav1.5 channels

Channels were studied in HEK293T cells using whole cell voltage clamp as described in the Materials and Methods.

(A) CiHv1 was studied using 1.5 s test pulses to +40 mV from a holding voltage of -60 mV with 10 s inter-pulse intervals. 1 μM $C6_2$ (red trace) was applied for 10 min and no apparent inhibition was observed compared to control currents measured before application (black trace).

(B) hKv1.3 was studied using 300 ms test pulses to +40 mV from a holding voltage of -80 mV with 10 s inter-pulse intervals. 1 μM $C6_2$ (red trace) was applied for 10 min and no apparent inhibition was observed compared to control currents measured before application (black trace).

(C) hNav1.5 was studied using 30 ms test pulses to 0 mV from a holding voltage of -100 mV with 10 s inter-pulse intervals. 1 μM $C6_2$ (red trace) was applied for 10 min and no apparent inhibition was observed compared to control currents measured before application (black trace).

(D) Effect of $C6_2$ (1 μM) on hHv1, CiHv1, hKv1.3 and hNav1.5 channels, showing peak currents at the end of the series of test pulses. Mean ± SEM; n = 3 cells for each channel.

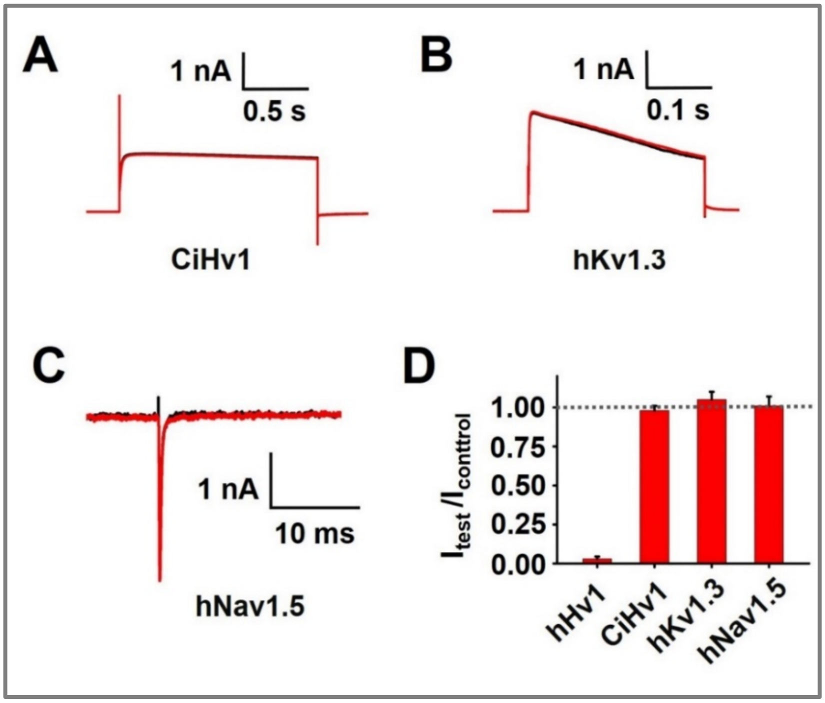

Supplemental Information Materials and Methods:

Inhibitor cysteine knot (ICK) phage display library construction and C6 toxin isolation.

As previously described, we designed a phage-display library of 1 million novel peptides sharing an ICK scaffold by combining elements of natural toxins (1). Phagemids expressing C6 were selected by their capacity to bind to hHv1 protein. Briefly, 110 ICK toxin sequences were selected from the UniProt database. Sequences were aligned based on the six conserved cysteine residues and were divided into A, B, and C domains defined by the second and fifth conserved cysteine. Nucleotide duplexes encoding A, B, and C domains were ligated unidirectionally to produce toxin variant genes with only the correct domain order. Separate reactions were preformed to ligate the gene inserts into pAS62 in frame with M13 filamentous phage particle coat protein pIII. Ligation mixtures were transformed into SS320 electrocompetent cells and sequencing performed to confirm unbiased insert utilization. Library phages were then prepared for screening as previously described (1).

hHv1 protein (hHv1-VSD, residues 75–223) was purified, biotinylated and adsorbed to streptavidin MagneSphere paramagnetic particles (1). Phage expressing the toxin library were mixed with hHv1 protein adsorbed to the Magnesphere beads and allowed to bind for 1 hour at room temperature. Phage toxin selection was performed using A KingFisher (ThermoFisher Scientific) automated magnetic bead manipulator. After 3 to 5 washes in binding buffer, tightly bound phage were detached from the beads using 100 mM dithiothreitol (DTT). The supernatant was collected and used immediately to infect log-phase *E. coli* XL-1 blue (Stratagene). Infected *E. coli* were used for quantification by tittering and for phage amplification. Alternative rounds of panning were done with NUNC-Immuno MaxiSorp 96-well plates (Thermo Fisher Scientific) that were coated 1 μg/well hHv1 protein to decrease non-specific phage enrichment seen using a single panning method. Phages were allowed to bind for 1 h at room temperature. Poorly adherent phage were removed by washing and bound phage were eluted with 100 μL of 0.1 M triethylamine by incubation for 10 min on a rocking shaker. The pH of the eluate was adjusted to between 7.0 and 8.5 with 1M Tris·HCl (pH 8.0). Amplification and tittering of

phage were as previously described (1). After five rounds of library sorting (three rounds using MagneSphere beads with adsorbed biotinylated hHv1 protein, and two rounds using NUNC-Immuno plates directly coated with hHv1 protein), clones were genotyped by DNA sequencing and enrichment of the C6 phagemid observed.

Molecular Biology

Human Hv1 (NM_001040107) tagged with a teal fluorescent protein (TFP) connected by a 13-residue flexible linker was constructed using gBlock gene fragments (Integrated DNA Technologies) and inserted into a laboratory dual purpose vector pMAX+ using Gibson Assembly (New England BioLabs), as reported (1). Per our prior reports (2), T-C6 was constructed by replacing the sequence of lynx1, a natural tethered nicotinic acetylcholine receptor peptide modulator, by the sequence of C6 (with N-terminus at front) in frame between the trypsin secretory signal sequence and a six residues flexible linker containing Gly-Asn repeat. The sequence encoding the hydrophobic chain of lynx1 for GPI attachment was inserted after the flexible linker. The whole sequence of T-C6 was cloned into the pCS2+ plasmid vector that has an SP6 promoter for *in vitro* transcription of cRNA. T-C6 with different length linkers were constructed using gBlock gene fragments (Integrated DNA Technologies) and Gibson Assembly (New England BioLabs), for instance, T-$C6_{46}$ has a 46 residues flexible linker in which a c-Myc epitope tag is incorporated. T-$C6_2$ was constructed in the backbone of T-C6 by replacing the C6 sequence with nucleotides encoding two C6 peptides linked by a flexible linker of either 5 or 20 Gly-Asn repeats using gBlocks. Point mutations of hHv1 and T-C6 were introduced using the QuikChange Site-Directed Mutagenesis Kit (Agilent). The sequences of all constructs were confirmed by DNA sequencing. Because hHv1 with V187C, L189C and L203C mutations did not have expression in HEK293T cells, alanine mutations were studied at these three sites.

Toxin Peptide Synthesis and Purification

C6 peptide (GenBank: AZI15804) and its variants, including C6-extN and C6-extC, were synthesized by CSBio. Peptide folding reactions were quenched by acidification and the peptides purified by HPLC, as before (1). Peptides that were more than 90% pure were lyophilized and stored at −20 °C. Sortase A was purchased from Sigma (SRP0676). Peptide ligation to create $C6_2$ was performed in a buffer with 50 mM Tris, 150 mM NaCl, and 10 mM $CaCl_2$ at pH 8, 60 μM C6-extN, 180 μM C6-extC, and 20 μM Sortase A. The mixture was incubated at 37 °C for 30 min and $C6_2$ purified using HPLC with a 0–100% gradient of solvent B versus solvent A in 50 min at 1 mL/min, where A is water with 0.1% trifluoroacetic acid (TFA) and B is acetonitrile with 1% TFA. UV detection was at 214 nm. Peptides were dissolved in external solutions for electrophysical recordings before use.

Cell Culture

HEK293T cells were purchased from ATCC and maintained in Dulbecco's Modified Eagle Medium (DMEM) (ATCC) supplemented with 10% fetal bovine serum and 1% penicillin and streptomycin. Plasmids were transfected into cells using Lipofectamine 2000 (Life Technologies) according to the manufacturer's instructions. Experiments were preformed 24 hours post transfection.

Whole-cell patch-clamp

Proton currents passed by hHv1 were recorded in whole cell mode using an Axopatch 200B amplifier. Stimulation and data collection were done with a Digidata1322A and pCLAMP 10 software (Molecular Devices). Cells were perfused with an external solution comprised of 100 mM HEPES, 100 mM NaCl, 10 mM glucose at pH 7.5. Pipettes with resistances between 3-5 MΩ were filled with 100 mM Bis-Tris buffer, 100 mM NaCl, and 10 mM glucose at pH 6.5. Capacitance was subtracted online. Sampling frequency was 10 kHz with filtering at 1 kHz. C6 block and unblock were monitored by evoking currents from a holding voltage of -60 mV with steps to a desired test voltage for 1.5 s with a 10 s

interpulse interval. Current-voltage relationships were similarly evoked from a holding potential of -60 mV to test pulses from -60 mV to 40 mV for 1.5 s in 20 mV intervals every 10 s. Fractional unblocked current was assessed at the end of the test pulse. The conductance-voltage relationships were determined as described by DeCoursey in (5) and were fit to the Boltzmann equation, $G = G_{max}/[1 + \exp(-zF(V-V_{1/2})/RT)]$, where V is the test potential, $V_{1/2}$ is the voltage of half-maximal activation, z is the effective valence, T is the temperature, R is the gas constant, and F is the Faraday constant. The dose response curves were determined by plotting the fractional unblocked current, $I_{toxin}/I_{control}$, versus concentration of toxins. Dose response curve was fit with Hill equation (Eq. 1) in Origin 8.0.

Two Electrode Voltage Clamp

Xenopus laevis stage VI oocytes were selected based on their size and clear white equatorial bands and injected with 10 ng of cRNA encoding hHv1 in the laboratory pMAX+ vector, as before (2, 6). pMAX+ carries 5' and 3' portions of the *Xenopus laevis* β-globin gene and a T7 promoter for *in vitro* transcription. To study the blocking effect of T-C6 (or variants), cRNAs for T-C6 (or variants) and hHv1 were mixed and co-injected into the oocytes. Recording solution was 60 mM NaCl, 1 mM $MgCl_2$, 2 mM $CaCl_2$, 120 mM HEPES, 40 mM sucrose at pH 7.2. To prevent changes in intracellular pH due to the proton efflux, oocytes were injected with 50 nL 1 M HEPES (pH = 7.2), to produce ~100 mM HEPES in the cytosol, 30 min before recording. Recordings were performed with constant gravity flow of solution at 2 mL/min yielding chamber exchange in under 5 s. Currents were recorded 2 days after cRNA injection using an Oocyte clamp amplifier OC-725C (Warner Instruments) with electrodes filled with 3 M KCl and resistances of 0.3 - 1 MΩ. Data were filtered at 1 kHz and digitized at 20 kHz using pClamp software and assessed with Clampfit v9.0. The fractional blockade by T-C6 was determined from the mean I_{toxin} with T-C6 (or variants) cRNA co-injection normalized to the mean $I_{control}$ with only channel cRNA injection.

Enzyme-linked Immunosorbent Assay

Surface expression of T-toxins were quantitated using ELISA as previously described (2). Oocytes injected with cRNAs of T-C6 variants bearing the c-Myc tag were blocked for 1 hour in recording solution with 3% BSA and then bound with c-Myc–Tag monoclonal antibody HRP (1 µg/mL) (Invitrogen) for 1 h. Oocytes were extensively washed using TEVC recording solution with 0.1% Tween 20 and transferred to recording solution without BSA and Tween 20. Individual oocytes were placed in V-bottom 96-well plate wells (Thermo Fisher Scientific) with 50 µL of 1-Step Ultra TMB-ELISA solution (Thermo Fisher Scientific) and incubated for 15 min. The reaction was stopped by adding 50 µL of 2 M H_2SO_4. Surface ELISA signals were quantitated at 450 nm using an Epoch Microplate Reader (BioTek).

Toxin Depletion Assay

1-palmitoyl-2-oleoyl-sn-glycero-3-phosphocholine (POPC) and 1-palmitoyl-2-oleoyl-sn-glycero-3-phosphoglycerol (POPG) were dried from a chloroform solution under a nitrogen stream. The dried lipids were rehydrated in a buffer comprised of 10 mM HEPES, 150 mM NaCl, 2 mM $CaCl_2$, 1 mM EDTA at pH 7.5. The resulting dispersions were extruded through 100 nm pore size polycarbonate filters (Millipore) and mixed at POPC:POPG = 3:1 ratio to form large unilamellar vesicles (LUVs). Varying concentrations of POPC:POPG LUVs or POPG only LUVs were added to an aqueous C6 solution (final concentration of C6 was 50 µM in 200 µL) and incubated with gentle agitation for 30 min at room temperature. For depletion experiments with native cell membranes, 170 oocytes and 8.5×10^6 HEK293T cells were used for incubation in a final volume of 400 µL. We estimated that the outer leaflet of an oocyte contains 0.15 nmol of lipid according to previous report (3). The same amount of lipid can be provided by 50,000 HEK293T cells, based on an average cell capacitance of ~10 pF, a specific capacitance of 1 $\mu F/cm^2$, and a surface area per lipid of 50 $Å^2$ (3). LUVs were separated by high-speed centrifugation (100,000 g, 20 min). Oocytes were separated by decantation, and HEK293T cells were separated by low-speed centrifugation (300 g, 4 min). C6 toxin remaining in the aqueous phase was determined using HPLC with a C18 column. C6 was eluted with a linear

gradient of 0–70% mobile phase B over 50 min at 1 mL/min (A was 0.1% TFA in water and B was 0.1% TFA in acetonitrile).

Simulation Systems and Molecular Dynamics Simulations

The homology model of C6 was constructed with the MODELLER program (7) using the NMR structure of the natural inhibitor cysteine knot (ICK) spider toxin Mu-agatoxin-Aa1a as the template (PDB: 1EIT) because it had the highest identity and sequence similarity with C6 on the "SWISS-MODEL" server. PDB file 1EIT includes 10 NMR models (**Fig. S5B**). Structural alignment and root mean square fluctuation (RMSF) analysis of the 10 structures show that the ICK scaffold is very stable (**Fig. S5B, C**), and shares high topological similarity with other ICK toxins (**Fig. S5D-F**), whereas loop regions 2 and 4 have higher conformational variability. We tested both the multiple templates module and the single template module of MODELLER, and because the multiple templates module using all 10 NMR models did not increase the model quality based on the model quality evaluation using the DOPE (Discrete Optimized Protein Energy) tool of MODELLER, we used only the last NMR model as the template. Comparing the C6 model and other ICK toxins demonstrated that the ICK scaffold of C6 had been well modelled (**Fig. S5D-F**). The bilayers of POPC:POPG (3:1) or pure POPC lipids were built using the web service CHARMM-GUI (8). The six cysteine residues of C6 were patched to form three disulfide bonds (C4-C18, C11-C23, and C17-C34) using the psfgen plugin of VMD (9). The C6 was placed on the extracellular side of the bilayer, and then solvated with 100 mM KCl solution using VMD. The two final C6-membrane systems were electrically neutral and contained ~59,000 atoms. The systems were minimized for 5,000 steps and equilibrated for 20 ns using NAMD (10), and then simulated for 1 μs on the special purpose computer Anton2 (11). The starting system of the C6-hHv1 complex was constructed using the equilibrated C6 structure and the transmembrane region (residues: G90 to I216) of the resting state dimeric hHv1 model adopted from our previous study (12) using VMD. Briefly, we used crystallographic structures of Ci-VSD to build an earlier dimeric hHv1 model (12) given the high sequence similarity between the two proteins and because the "down" state of Ci-VSD formed a dimer in the crystallographic condition (12). To-date, two

experimentally determined structures of Hv1 proton channel have been deposited (**Fig. S6A-B**): the crystallographic structure of a trimeric chimera mouse proton channel, mHv1cc (PDB: 3wkv) (13), and the NMR structures of a monomeric human proton channel, hHv1 (PDB: 5oqk) (14). The NMR models provide consistent secondary structures of hHv1; however, their tertiary structures vary considerably from each other and all other available structures of canonical voltage sensors. We updated the secondary structure of our earlier homology model (12) by changing D123-I126 at the C-terminal end of S1 and Q194-E196 at the N-terminal end of S4 from loop into helical conformation based on the NMR structures of hHv1. Helices S1 and S4 of our homology model form the dimer interface at the extracellular and intracellular sides, respectively, ensuing that the continuation of the S4 helices can form the coiled-coil structure (**Fig. S6F-H**). A monomeric model of hHv1 has recently been provided in the AlphaFold database (see https://alphafold.ebi.ac.uk/entry/Q96D96) (15, 16). Superimposition of the AlphaFold model and one subunit of our dimeric homology model (**Fig. S6C**) shows that the two are quite similar with modest backbone RMSDs of transmembrane helices S1 (1.2 Å), S2 (3.2 Å), and S4 (2.6 Å). In contrast, the AlphaFold model places the helix S3 one helix turn further inward resulting in a notable conformation change with a RMSD of 7.9 Å (**Fig. S6C**). hHv1 homology model was inserted into a bilayer of POPC:POPG (3:1) lipids, and two C6 molecules were placed on the extracellular side of hHv1 with the F28 containing loop (loop 4) facing the lipids and the potential binding interface facing the S3-S4 loop of each monomer of the channel. The system was then solvated with 100 mM KCl solution and kept electrically neutral. The final system contained ~115,000 atoms with an orthorhombic periodic box of ~96 × 96 × 120 Å^3. A stepwise target MD simulations protocol (2) was used to refine the complex with distance restraints between C6-lipids and C6-hHv1 using NAMD. The system was minimized for 5,000 steps and equilibrated for 50 ns with positional restraints of 1 kcal/mol/Å^2 being applied to the toxin and protein backbone atoms to relax the lipids. Then the positional restraints were removed except those applied on S4 backbone atoms of hHv1 for maintaining the dimeric interface because the cytosolic coiled-coil structure of hHv1 was not included in the model to decrease the system size. The C6 molecules were initially pulled vertically towards the lipids by a distance restraint between the Cα atom of F28 of C6 and the center-of-mass

of the phosphate atoms of the lipids using the Colvars module (17) for 10 ns. Then two additional distance restraints were added in the subsequent 140 ns simulation to accelerate the binding of C6 to hHv1: the distance between the sidechain heavy atoms of C6-F28 and the Cγ atom of hHv1-L200 and the distance between the sidechain heavy atoms of C6-M33 and the Cα atoms of hHv1-H193.

During the last 100 ns simulation, three pairs of distance restraints were applied: the distance between the sidechain heavy atoms of C6-F28 and the Cγ atom of hHv1-L200, the distance between the sidechain heavy atoms of C6-W38 and the Cα atom of hHv1-V187, and the distance between the sidechain heavy atoms of C6-M33 and hHv1-H193. A target distance of 4 Å was used for the pairwise distant restraints and the force constant gradually decreased from 10.0 to 0.1 kcal/mol/Å^2. In addition, a C2 symmetry restraint was applied to the two C6 molecules, a distance restraint was applied between C6-R35 and C6-S7/E19, and a RMSD restraint was applied to each monomer of hHv1 during the simulations. The system was simulated for another 50 ns with all distance restraints removed.

The "up" state of the hHv1 model was built by moving the S4 helix of the "down" state one helical turn outward, while leaving the S1-S3 helices unchanged as in our previous study (12). The $C6_2$ model was built by connecting the two equilibrated C6 molecules with a linker of 10 residues (LPATGGGGGG) using MODELLER and equilibrated for 10 ns with positional restraints being applied to the two C6 peptides. The $C6_2$ model was then inserted into the C6-hHv1 system to replace the two C6 monomer. The $C6_2$-hHv1 system was then simulated for 150 ns with the same distance restraints being applied as in the C6-hHv1 system in the first 100 ns and no distance restraints in the last 50 ns. A C2 symmetry restraint and a RMSD restraint were also applied to the two C6 molecules in $C6_2$ and each monomer of hHv1, respectively during the simulations.

All the MD simulations were carried out under the NPT ensemble (constant temperature at 300 K and constant pressure at 1 atm) in the periodic boundary conditions with a time step of 2 fs. The CHARMM36 force field parameter set with torsional backbone corrections (18) was used for proteins, lipids and ions, and the TIP3P model for water molecules (19). In NAMD simulations, the Langevin dynamics and the Nose−Hoover

Langevin piston method (20, 21) were used to control temperature and pressure, respectively. The long-range electrostatic interaction was calculated using the particle mesh Ewald (PME) method (22), and the van der Waals interaction was smoothly switched off at 10−12 Å. In Anton2 simulations, the Nose–Hoover thermostat and the semi-isotropic MTK barostat (20, 23) were used to control temperature and pressure, respectively. The long-range electrostatic interaction was calculated using the k-space Gaussian split Ewald method (24).

Supplemental Information References

1. R. Zhao *et al.*, Role of human Hv1 channels in sperm capacitation and white blood cell respiratory burst established by a designed peptide inhibitor. *Proc Natl Acad Sci U S A* **115**, E11847-E11856 (2018).
2. R. Zhao, H. Dai, N. Mendelman, J. H. Chill, S. A. N. Goldstein, Tethered peptide neurotoxins display two blocking mechanisms in the K(+) channel pore as do their untethered analogs. *Sci Adv* **6**, eaaz3439 (2020).
3. M. Milescu *et al.*, Tarantula toxins interact with voltage sensors within lipid membranes. *J Gen Physiol* **130**, 497-511 (2007).
4. H. Xu *et al.*, Structural Basis of Nav1.7 Inhibition by a Gating-Modifier Spider Toxin. *Cell* **176**, 702-715 e714 (2019).
5. B. Musset, T. Decoursey, Biophysical properties of the voltage gated proton channel H(V)1. *Wiley interdisciplinary reviews. Membrane transport and signaling* **1**, 605-620 (2012).
6. R. Zhao *et al.*, Designer and natural peptide toxin blockers of the KcsA potassium channel identified by phage display. *Proc Natl Acad Sci U S A* **112**, E7013-7021 (2015).
7. A. Sali, T. L. Blundell, Comparative protein modelling by satisfaction of spatial restraints. *J Mol Biol* **234**, 779-815 (1993).
8. S. Jo, T. Kim, V. G. Iyer, W. Im, CHARMM-GUI: a web-based graphical user interface for CHARMM. *J Comput Chem* **29**, 1859-1865 (2008).
9. W. Humphrey, A. Dalke, K. Schulten, VMD: visual molecular dynamics. *J Mol Graph* **14**, 33-38, 27-38 (1996).
10. J. C. Phillips *et al.*, Scalable molecular dynamics with NAMD. *J Comput Chem* **26**, 1781-1802 (2005).
11. D. E. Shaw *et al.* (2014) Anton 2: raising the bar for performance and programmability in a special-purpose molecular dynamics supercomputer. in *Proceedings of the International Conference for High Performance Computing, Networking, Storage and Analysis* (IEEE Press, New Orleans, Louisana), pp 41–53.
12. Q. Li *et al.*, Resting state of the human proton channel dimer in a lipid bilayer. *Proc Natl Acad Sci U S A* **112**, E5926-5935 (2015).

13. K. Takeshita *et al.*, X-ray crystal structure of voltage-gated proton channel. *Nat Struct Mol Biol* **21**, 352-357 (2014).
14. M. Bayrhuber *et al.*, Nuclear Magnetic Resonance Solution Structure and Functional Behavior of the Human Proton Channel. *Biochemistry* **58**, 4017-4027 (2019).
15. J. Jumper *et al.*, Highly accurate protein structure prediction with AlphaFold. *Nature* **596**, 583-589 (2021).
16. M. Varadi *et al.*, AlphaFold Protein Structure Database: massively expanding the structural coverage of protein-sequence space with high-accuracy models. *Nucleic Acids Res* **50**, D439-D444 (2022).
17. G. Fiorin, M. L. Klein, J. Hénin, Using collective variables to drive molecular dynamics simulations. *Molecular Physics* **111**, 3345-3362 (2013).
18. A. D. MacKerell, Jr., M. Feig, C. L. Brooks, 3rd, Improved treatment of the protein backbone in empirical force fields. *J Am Chem Soc* **126**, 698-699 (2004).
19. W. L. Jorgensen, J. Chandrasekhar, J. D. Madura, R. W. Impey, M. L. Klein, Comparison of simple potential functions for simulating liquid water. *The Journal of Chemical Physics* **79**, 926-935 (1983).
20. G. J. Martyna, D. J. Tobias, M. L. Klein, Constant pressure molecular dynamics algorithms. *The Journal of Chemical Physics* **101**, 4177-4189 (1994).
21. S. E. Feller, Y. Zhang, R. W. Pastor, B. R. Brooks, Constant pressure molecular dynamics simulation: The Langevin piston method. *The Journal of Chemical Physics* **103**, 4613-4621 (1995).
22. U. Essmann *et al.*, A smooth particle mesh Ewald method. *The Journal of Chemical Physics* **103**, 8577-8593 (1995).
23. G. J. Martyna, M. L. Klein, M. Tuckerman, Nosé–Hoover chains: The canonical ensemble via continuous dynamics. *The Journal of Chemical Physics* **97**, 2635-2643 (1992).
24. Y. Shan, J. L. Klepeis, M. P. Eastwood, R. O. Dror, D. E. Shaw, Gaussian split Ewald: A fast Ewald mesh method for molecular simulation. *J Chem Phys* **122**, 54101 (2005).
